# Mrgprb2-mediated mast cell activation exacerbates Modic changes by regulating immune niches

**DOI:** 10.1038/s12276-024-01230-1

**Published:** 2024-05-01

**Authors:** Zhongyin Ji, Jie Li, Siyue Tao, Hui Li, Xiangxi Kong, Bao Huang, Zhenhua Feng, Xiaoan Wei, Zeyu Zheng, Jian Chen, Binhui Chen, Junhui Liu, Fengdong Zhao

**Affiliations:** 1 Department of Orthopaedic Surgery, Sir Run Run Shaw Hospital, Zhejiang University School of Medicine, No. 3, Qingchun Road East, Hangzhou, 310016 P. R. China; 2 Key Laboratory of Musculoskeletal System Degeneration and Regeneration Translational Research of Zhejiang Province, No. 3, Qingchun Road East, Hangzhou, 310016 P. R. China; 3 Department of Orthopaedic Surgery, Ningbo Medical Center Li Huili Hospital, Ningbo, Zhejiang China

**Keywords:** Cartilage, Mast cells

## Abstract

Modic changes are radiographic features associated with microfracture, low-virulence organism infection and chronic inflammation with inflammatory cell infiltration in the vertebral endplate region. Mast cells, as innate immune cells similar to macrophages, are present in painful degenerated intervertebral discs. However, the involvement and mechanisms of mast cells in the development of Modic changes remain unclear. Herein, we found increased mast cell infiltration in samples from patients with Modic changes and in mouse models of Modic changes. To clarify the role of mast cells in the progression of Modic changes, we used mast cell-deficient (KIT^W-SH/W-SH^) mice to construct a model of Modic changes and found that the severity of Modic changes in KIT^W-SH/W-SH^ mice was significantly lower than that in WT mice. These findings were further supported by the use of a mast cell-specific activator (compound 48/80) and a stabilizer (cromolyn). Furthermore, we found that mast cells were not activated via the classic IgE pathway in the Modic change models and that Mrgprb2 is the specific receptor for mast cell activation reported in recent studies. Then, we utilized Mrgprb2 knockout mice to demonstrate that Mrgprb2 knockout inhibited mast cell activation and thus reduced the degree of Modic changes. Transcriptomic sequencing revealed aberrant PI3K-AKT and MAPK pathway activation in the Mrgprb2-deficient mast cells. Additionally, Mrgpbrb2-activated mast cells regulate immune niches by recruiting macrophages, promoting M1 polarization and reducing M2 polarization, thereby promoting the progression of Modic changes. These findings suggest that mast cells may serve as a novel therapeutic target for addressing Modic changes.

## Mast cells drive modic changes progression via Mrgprb2 activation

Degenerative disorders of the lower back are common and can greatly affect life quality. The research by Ji et al. examines the role of mast cells (a kind of immune cell) in the development of these disorders. They discovered that mast cells aid in the progression of Modic changes (a type of spinal degeneration), by affecting the metabolism of the endplates (the ends of the spinal bones) and altering the immune response. This was shown through experiments with human tissue samples, animal models, and cell studies. The results suggest that focusing on mast cells could be a potential treatment for Modic changes. The research also emphasizes the significance of the Mrgprb2 receptor (a protein that activates mast cells) as a potential drug target. “This summary was initially drafted using artificial intelligence, then revised and fact-checked by the author.”

## Introduction

Degenerative disorders of the lumbar spine are characterized by a high prevalence and prolonged duration^1,2^. The etiology of these disorders is multifactorial^3,4^. The clinical presentation typically involves low back pain, with or without radiation to the lower extremities, which substantially impacts quality of life for affected individuals^5^. Moreover, these disorders have increasingly been occurring in younger age groups^6,7^. The prevalence of lumbar degenerative diseases is anticipated to increase in tandem with the aging of society, resulting in a substantial societal burden. An increased number of patients have undergone surgery, and increasing numbers of patients have been diagnosed with Modic changes by magnetic resonance imaging (MRI).

Modic changes are distinct manifestations of intervertebral disc degenerative disease (IVDD) and are characterized by alterations in MRI signals within the vertebral endplate region^8–10^. Modic changes are commonly observed in the lower lumbar spine^11,12^. These changes are strongly linked to lumbar degenerative diseases and low back pain^13,14^, as they affect the mechanical properties of the intervertebral disc and endplate, leading to a poor response to conservative treatment^15–17^. The pathophysiology of Modic changes involves complex mechanisms, including mechanical injury, autoimmune reactions, low-grade infections, and inflammatory responses^18–20^. These factors promote the formation of inflammatory immune niches in the endplate region, where inflammatory cells such as macrophages infiltrate^21^. However, the specific cellular immune mechanism involved in Modic changes is unclear, especially the role of mast cells, which has not been studied.

Derived from hematopoietic stem cells in the bone marrow, mast cells are widely distributed in the bloodstream and mucosal tissues and exhibit multifaceted functions beyond immune surveillance^22,23^. Upon stimulation by pathogens or allergens, mast cells rapidly activate and undergo degranulation to release various bioactive molecules, including histamine, leukotrienes, chemokines, and tryptases^24,25^. Furthermore, mast cells can secrete proinflammatory cytokines, including TNF, IL-4, IL-6, and IL-33^26,27^, which play crucial roles in regulating immune responses. Previous studies have shown that FcεRI receptors expressed on mast cells can be activated by IgE and subsequently undergo degranulation and proinflammatory cytokine release, leading to type I hypersensitivity^28,29^. Recent studies have shown that Mrgprb2, also known as MRGPRX2 in humans, is a specific receptor that triggers mast cell activation independently of IgE-FcεRI in mice^30,31^. Activation of Mrgprb2 triggers altered mediator release, including increased tryptase secretion and decreased monoamine release, leading to the generation of nonhistamine substances and pruritus^32,33^. Recent studies have demonstrated the involvement of mast cells in intervertebral disc degeneration^34^. The potential involvement, activation and downstream targets of mast cells in the pathogenesis of Modic changes require further investigation.

This study focused on investigating the activation pathways of mast cells and their interactions with macrophages and chondrocytes in Modic changes. These findings highlight the potential therapeutic approach of targeting mast cells to alleviate Modic changes.

## Materials and methods

### Patient tissue samples

This study involved the collection of human intervertebral disc tissue samples from patients who underwent lumbar disc herniation surgery. The Ethics Committee of the Sir Run Run Shaw Hospital approved this research, and informed consent was obtained from all patients or their relatives prior to surgery. We categorized the collected samples into two groups: one group consisted of patients with Modic changes, while patients without Modic changes served as the control group. The tissue samples were fixed in 4% paraformaldehyde at 4 °C for 48 h for further experiments.

### Mice

Mrgprb2 global knockout mice were obtained from Gempharmatech (China), while KIT^W-SH/W-SH^ mice were kindly provided by Professor Linrong Lu from Zhejiang University. The transgenic mice utilized in this research were generated on a C57BL/6 pure strain and maintained under specific pathogen-free conditions. The housing conditions were a controlled environment with a temperature of ~22 °C and a light/dark cycle of 12 h each^21,35^. Wild-type littermates of the same sex were chosen as the control group for all the experiments. The animal studies conducted in this research received ethical approval from the Ethics Committee of Sir Run Run Shaw Hospital.

### Preparation of *Cutibacterium acnes* supernatant

Cultures of *C. acnes* (ATCC 6919) were prepared by incubating the bacteria in 15% tryptic soy broth (TSB) under anaerobic conditions at ~37 °C for 2 weeks. After the incubation period, the bacteria were subjected to centrifugation at 4000 × *g* for 5 min and then reconstituted in phosphate-buffered saline (PBS) for a concentration of 1 × 10^7^ CFU/ml. For anaerobic conditions, the bacteria were cultured in an oxygen-free environment using an anaerobic bag at 37 °C for an additional 2 days. Subsequently, the bacteria were subjected to centrifugation at 4000 × *g* for 20 min, after which the resulting supernatant was harvested. The supernatant was filtered twice using a 220 nm molecular sieve.

### Isolation and in vitro culture of murine mast cells

Male C57BL/6 mice (8 weeks) were euthanized by cervical dislocation and sterilized with a 5-min alcohol soak. The hind limbs, including the femur and tibia, were isolated, and the bone marrow was flushed out using 1640 medium supplemented with 10% fetal bovine serum (FBS). The cell mixture was then seeded into a 75 cm^2^ cell culture flask supplemented with 10 ng/ml stem cell factor (SCF) and 30 ng/ml interleukin-3 (IL-3). The cell culture flask was placed in a CO_2_ incubator set at 37 °C with a 5% CO_2_ atmosphere. The culture medium was refreshed every 3–4 days. The floating cells were cultured for a period of 4–6 weeks. The purity of the mast cells was assessed using toluidine blue staining and flow cytometry.

### Murine chondrocyte extraction

Knee cartilage was obtained from 5-day-old C57BL/6 suckling mice immediately after euthanasia. The cartilage tissues were rinsed with salt solution (Hank’s, Gibco, Grand Island, NY, USA) and then fragmented. Subsequently, the fragments were digested for 24 h using 0.2% type-2 collagenase (Sigma, USA). The next day, the mixture was filtered through a 100 μm cell strainer. After two washes with Hank’s solution, the isolated cells were cultured in a 5% CO_2_, 37 °C incubator using complete culture medium comprising DMEM (Gibco, Invitrogen, USA) supplemented with 10% FBS (Gibco, Invitrogen, USA) and antibiotics. The culture medium was replenished every 2–3 days.

### RNA isolation and quantitative RT‒PCR

Total RNA was extracted from the different groups using TRIzol reagent (Invitrogen). A NanoDrop 2000 spectrophotometer was used to measure the concentration and purity of the RNA samples. The RNA samples were subjected to reverse transcription using PrimeScript RT MasterMix (TaKaRa Bio, Japan), which facilitated the synthesis of complementary DNA (cDNA). The mRNA levels were assessed using quantitative real-time polymerase chain reaction (RT‒qPCR) with SYBR Green qPCR Master Mix (TaKaRa Bio, Japan). The RT‒qPCRs consisted of an initial denaturation step at 95 °C for 5 min, followed by 40 cycles of amplification (95 °C for 15 s and 60 °C for 60 s), and a final melting curve analysis (95 °C for 15 s and 60 °C for 60 s). The RT‒qPCR experiments were conducted in triplicate to ensure reproducibility and statistical reliability. The mRNA expression levels were normalized to those of the internal control gene β-actin. The sequences of primers used in the experiments are provided in Supplementary Table 1.

### Western blotting

Protein extraction was performed by treating the samples with RIPA lysis buffer supplemented with phosphatase and protease inhibitors (Beyotime, China). SDS‒PAGE was used to separate the resulting proteins. Subsequently, the proteins were transferred onto PVDF membranes for subsequent analysis. After the PVDF membranes were blocked with 5% bovine serum albumin (BSA) at room temperature for 60 min, they were cut appropriately according to the molecular weight of the target proteins and incubated for 12 h with primary antibodies. The primary antibodies used included antibodies against Aggrecan (#ab3778, Abcam, UK, 1:1000), SOX9 (#ET1611-56, Huabio, China, 1:1000), Col2a1 (#ab307674, Abcam, UK, 1:1000), MMP3 (#ab52915, Abcam, UK, 1:1000), MMP13 (#ab219620, Abcam, UK, 1:1000), Adamts5 (#ab41037, Abcam, UK, 1:1000), PI3K (#ab191606, Abcam, UK, 1:1000), p-PI3K (#ab182651, Abcam, UK, 1:1000), AKT (#ab179463, Abcam, UK, 1:1000), p-AKT (#ab192623, Abcam, UK, 1:1000), p65 (#ab16502, Abcam, UK, 1:1000), p-p65 (#ab76302, Abcam, UK, 1:1000), p38 (#ab170099, Abcam, UK, 1:1000), p-p38 (#ab4822, Abcam, UK, 1:1000), JNK (#ab179461, Abcam, UK, 1:1000), p-JNK (#ab124956, Abcam, UK, 1:1000), Erk (#ab184699, Abcam, UK, 1:1000), p-Erk (#ab201015, Abcam, UK, 1:1000), Mrgprb2 (#PA5-113198, Invitrogen, USA, 1:1000), tryptase (#ET161064, Huabio, China, 1:1000), and β-actin (#AF2811, Beyotime, China, 1:1000). The membranes were incubated with HRP-linked secondary antibodies, including anti-rabbit IgG HRP-linked antibody (#7074S, CST, USA, 1:5000) and anti-mouse IgG HRP-linked antibody (#7076S, CST, USA, 1:5000). The protein bands were visualized using chemiluminescence reagents provided by Amersham Biosciences (Buckinghamshire, USA).

### Safranin O & Fast Green staining

Mouse tails were dissected to obtain specimens comprising vertebra-disk-vertebra. These specimens were subjected to fixation in 4% paraformaldehyde (4 °C, 48 h) and subsequently decalcified using EDTA for 14 days. Subsequently, the specimens were subjected to sequential dehydration, paraffin embedding, and sectioning into 5-μm-thick slices. For preparation of the sections for histology and immunohistochemistry, the sections were deparaffinized with xylene, rehydrated using solutions of various concentrations of ethanol, and subsequently stained using standard protocols. Staining techniques such as hematoxylin and eosin (H&E), Alcian blue, and Safranin O/Fast Green were used to visualize specific tissue features.

### Immunohistochemistry

The sections were treated with H_2_O_2_ (3%) for 20 min to block endogenous peroxidase activity. Subsequently, trypsin incubation was performed for 20 min, followed by blocking of nonspecific antigens using a solution containing 1% Tween-20 and 5% bovine serum albumin in PBS for 60 min. The sections were incubated with primary antibodies overnight at 4 °C. The antibodies used included Sox9 (#ET1611-56, Huabio, China, 1:200), MMP13 (#ab219620, Abcam, UK, 1:200), and tryptase (#ET1610-64, Huabio, China, 1:200). Corresponding secondary antibodies conjugated to HRP (CST, 1:5000) were then applied to the sections. Subsequently, the sections were counterstained with hematoxylin. Quantification of positive cells was conducted by capturing images of 6 random fields at a magnification of 100× using ImageJ software. Cell quantification was performed independently by three researchers, and the results were averaged based on a minimum of three sections from each specimen.

### Immunofluorescence staining

The sections were blocked with 5% BSA for 60 min, followed by a 12 h incubation at 4 °C with primary antibodies against Col2 **(**#ab307674, Abcam, UK, 1:200), F4/80 (#27076S, CST, USA, 1:200), CD86 (#91882S, CST, USA, 1:200), and CD163 (#ab87099, Abcam, UK, 1:200). After the sections were washed, they were incubated for 60 min at room temperature with anti-rabbit Alexa Fluor (488) secondary antibody (#710369, Invitrogen, USA, 1:300) and anti-rabbit Alexa Fluor (555) secondary antibody (#A31572, Invitrogen, USA, 1:300). DAPI (#D9542, Sigma-Aldrich, US) was used to stain the nuclei (0.1 μg/mL, 30 min, room temperature). Histological scoring and quantitative analysis of immunofluorescence staining were conducted in a double-blinded manner to minimize bias.

### Micro‐CT analysis

After fixation in paraformaldehyde (4%) for 48 h at room temperature, the caudal vertebral segments of the mice were imaged using a specific micro-CT scanner (model: Skyscan 1275, Aartselaar, Belgium). The imaging procedure utilized X-rays with parameters set at 60 μA/50 kV and 9 μm.

### Mouse model of Modic changes

Prior to surgery, the mice were anesthetized. With anteroposterior and lateral X-rays as a guide, the intervertebral space between the fourth and fifth caudal vertebrae was identified. A 26 G needle was then inserted perpendicularly to the vertebral body. Once the needle entered the intervertebral space, it was angled toward the middle region of the endplate of the proximal vertebral body at a 30° angle from the horizontal. Subsequently, 0.01 mL of *C. acnes* suspension (1 × 10^7^ CFU/mL, ATCC 6919, China) was injected. Acupuncture was performed in the endplate region of mice in the control group, without any injection of *C. acnes*.

### Cell scratch assay

A linear scratch was created on the cell monolayer of near-confluent RAW 264.7 cells using a 100 μl plastic pipette tip. The cells were cultured in 1640 medium without FBS, and a scratch was created through the cell monolayer using a pipette tip. The gap width was measured at 0 h and 24 h after scratching using an inverted microscope. The experiment was conducted in triplicate to ensure reliability and statistical significance.

### Macrophage migration assay

A Costar Transwell system (CLS3364, Corning) was used for the Transwell migration assay.

A total of 5 × 10^3^ iBMDMs were resuspended in serum-free medium (150 μl) and transferred to the upper chamber of the Costar Transwell system. Then, 350 μl of conditioned medium was added to the lower chamber. Following a 24 h incubation period, the cells that successfully migrated through the 8-μm pore membrane reached the lower chamber. Subsequently, the migrated cells were stained with crystal violet solution and observed by microscopy at 10× magnification for analysis.

### Statistical analysis

The data are presented as the mean ± standard deviation (SD) of individual data points. We performed statistical analysis using SPSS 19.0 (SPSS, Chicago) to assess the normality of the distribution of the data via the Shapiro‒Wilk test. For normally distributed data, we used Student’s t test, one-way ANOVA, and Tukey’s post hoc analysis to identify significant differences between groups. Nonparametric tests were used for non-normally distributed data. We considered a significance level of *P* < 0.05 to indicate statistical significance. To ensure robustness and reproducibility, we independently conducted all experiments at least three times.

## Results

### Mast cells are involved in the development of Modic changes

**Fig. 1:**
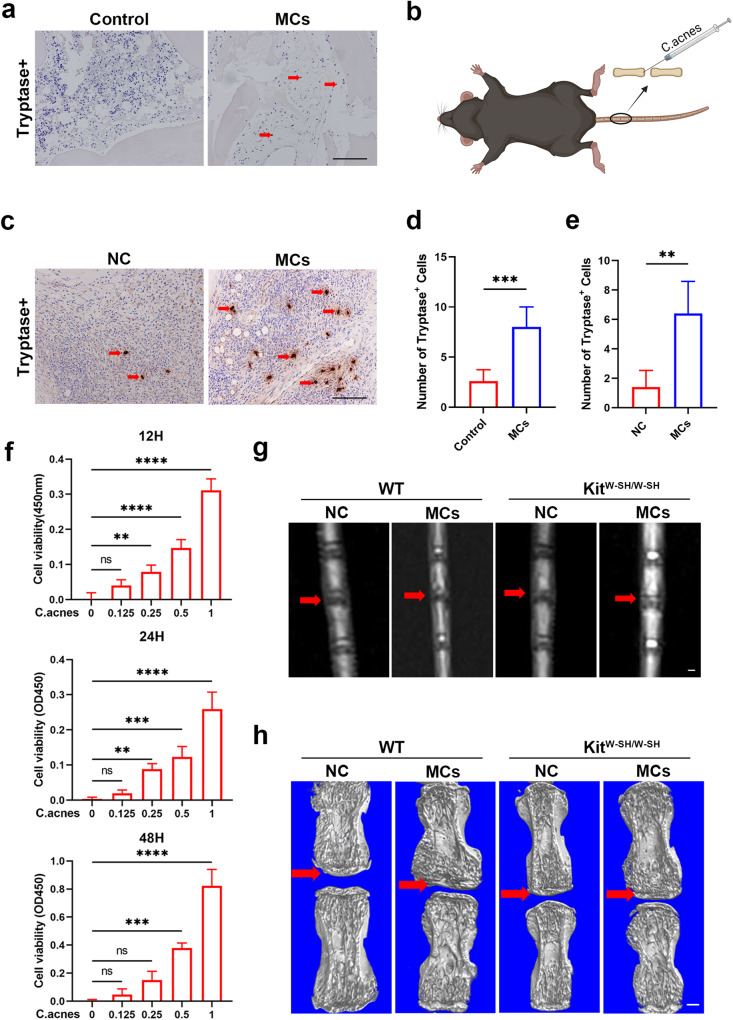
Mast cells are involved in the development of Modic changes. **a** Immunohistochemical staining of tryptase in human intervertebral disc sections with or without Modic changes (*n* = 4 per group). **b** Schematic diagram of the mouse model of Modic changes (*n* = 6 per group). **c** Immunohistochemical staining of tryptase in mouse intervertebral disc sections with or without Modic changes for 4 weeks (*n* = 6 per group). **d** Tryptase-positive cells were quantified by counting the number of cells per high-magnification field of view from human tissues (*n* = 4 per group). **e** Tryptase-positive cells were quantified by counting the number of cells per high-magnification field of view from mouse tissues (*n* = 6 per group). **f** Relative cell viability assays (CCK-8) of WT mast cells stimulated with *C. acnes* (10^7^ CFU/ml) (*n* = 4 per group). **g** Representative T2-weighted images of caudal discs from WT and KIT^W-SH/W-SH^ mice with or without Modic changes for 4 weeks (*n* = 6 per group). **h** Three-dimensional reconstructed micro-CT images from the above four groups (*n* = 6 per group). Scale bar, 200 μm. The data are presented as the mean ± SD. Student’s t test and one-way ANOVA with Tukey’s multiple comparison test were used for statistical analysis (*P < 0.05, **P < 0.01, ***P < 0.005, ****P < 0.001).

Modic changes refer to chronic inflammatory reactions in the vertebral endplate region, and the involvement of mast cells in this process has not been elucidated^36,37^. To investigate this issue, we analyzed human samples from patients with intervertebral disc degeneration diseases that were divided into two groups: the Modic change group and the non-Modic change group (as the control). Our findings indicated that tissues exhibiting Modic changes displayed more pronounced infiltration of mast cells than did those without Modic changes (Fig. 1a, d). Then, we generated a model of *C. acnes* infection-induced Modic changes (MCs) in the tail vertebrae of mice. In the MCs group, we observed a pronounced increase in mast cell infiltration compared to that in the NC group, which only received acupuncture (Fig. 1b, c, e). We speculated that mast cells contribute to the occurrence and progression of Modic changes.

Furthermore, experiments were performed to investigate the effect of *C. acnes* stimulation on mast cells in vitro. Given that *C. acnes* contributes to the induction of Modic changes, we cocultured the supernatants from *C. acnes* cultures with mast cells. CCK-8 analysis at three time points showed that mast cell activity was proportional to the supernatant concentration (Fig. 1f). Toluidine blue staining revealed that the volume of mast cells increased after stimulation with the supernatant, and the number of granules in the cytoplasm increased significantly (Supplementary Fig. 1a).

Subsequently, we utilized mast cell-deficient mice (KIT^W-SH/W-SH^) to model Modic changes^38^. After 4 weeks, the samples were examined using microcomputed tomography and MRI. After Modic changes were induced, the T2 signal in the MR images of the KIT^W-SH/W-SH^ group was greater than that in the MR images of the control group (Fig. 1g). Micro-CT revealed that KIT^W-SH/W-SH^ mice exhibited less bone loss in the endplate region and compensatory vertebral thickening after modeling (Fig. 1h). These findings demonstrated a decreased degree of Modic changes and suggested that mast cells play a pivotal role in the pathogenesis of Modic changes.

### Mast cells influence endplate matrix metabolism in regions with Modic changes

**Fig. 2:**
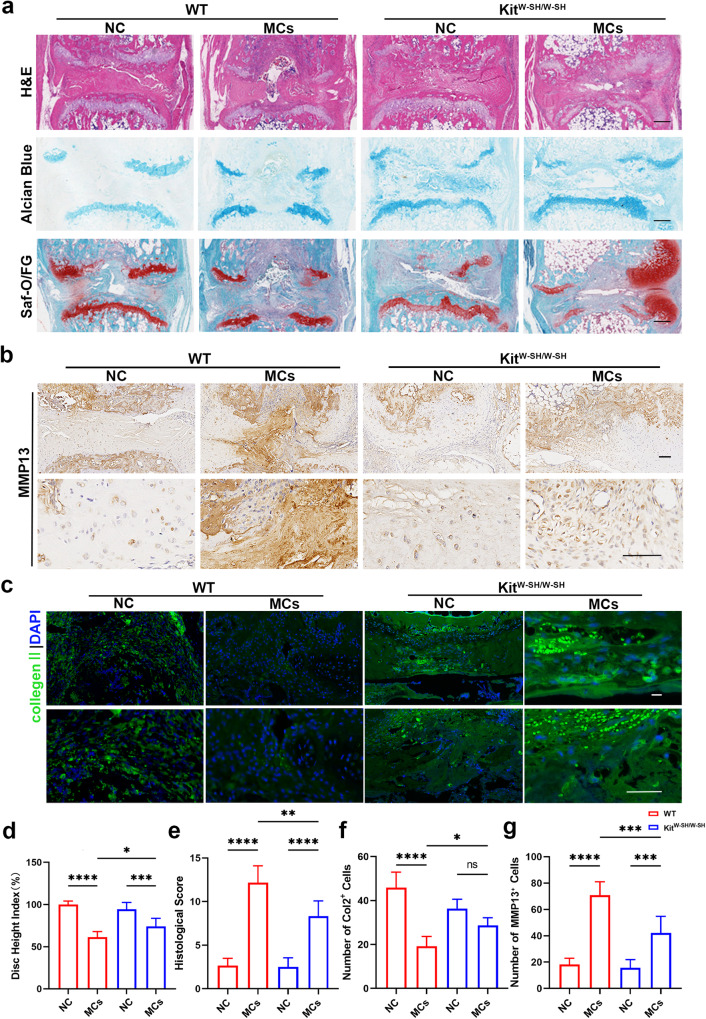
Mast cells influence endplate matrix metabolism in regions with Modic changes. **a** Representative images of H&E, Safranin O/Fast Green, and Alcian blue staining. **b** Immunohistochemistry (IHC) was used to determine the expression of MMP13. The results of the quantitative analysis are presented in (**f**) (*n* = 6 per group). **c** Immunofluorescence (IF) showing the expression of Col2. The results of the quantitative analysis are presented in (**g**) (*n* = 6 per group). **d** The disc height indices of the different groups (*n* = 6 per group). **e** Histological scores of the different groups (*n* = 6 per group). Scale bar, 50 μm. The data are presented as the mean ± SD, and one-way ANOVA with Tukey’s multiple comparison test was used for statistical analysis (*P < 0.05, **P < 0.01, ***P < 0.005, ****P < 0.001).

To gain a deeper understanding of the involvement of mast cells in Modic changes, we performed histological staining, including hematoxylin and eosin (H&E), Safranin O/Fast Green, and Alcian blue staining. Compared to the NC group, the WT MCs group exhibited disappearance of the nucleus pulposus, bilateral cartilage defects, and endplate destruction, while the KIT^W-SH/W-SH^ MCs group exhibited fewer cartilage defects, bone damage, and lesions involving only one side (Fig. 2a, d, e).

Furthermore, we conducted immunofluorescence and immunohistochemical staining for matrix metabolic proteins. In the WT MCs group, Col2 and Sox9 expression was obviously decreased, while MMP13 expression was elevated, and these changes were much greater than those in the KIT^W-SH/W-SH^ groups (Fig. 2b, c, f, g, Supplementary Fig. 1b, c). Based on the observed results, we concluded that mast cells play an important role in the development of Modic changes by influencing matrix metabolism in the region between the intervertebral disc and the endplate (cartilage and bony endplate).

### The activation of mast cells exacerbates Modic changes

**Fig. 3:**
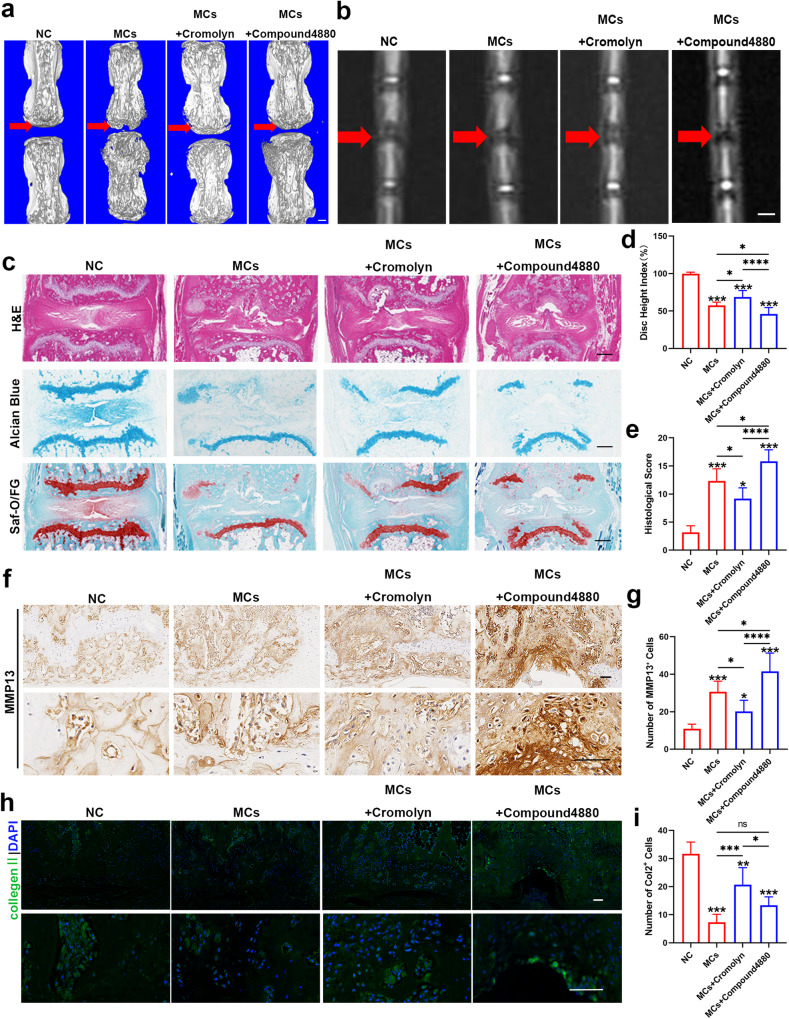
The activation of mast cells significantly exacerbated Modic changes. **a** Three-dimensional reconstruction of micro-CT images obtained from the NC, *C. acnes*, *C. acnes*+cromolyn, and *C. acnes*+compound 48/80 groups after 4 weeks (*n* = 6 per group). **b** T2-weighted images of caudal segments from WT mice in the NC, *C. acnes*, *C. acnes*+cromolyn, and *C. acnes*+compound 4880 groups were obtained after 4 weeks (*n* = 6 per group). Scale bar, 200 μm. **c** Representative images of H&E, Safranin O/Fast Green, and Alcian blue staining from different groups. **d** The disc height indices of the different groups (*n* = 6 per group). **e** Histological scores of the different groups (*n* = 6 per group). **f** Immunohistochemistry (IHC) was used to determine the expression of MMP13. The results of the quantitative analysis are presented in (**g**). Scale bar, 50 μm (*n* = 6 per group). **h** Immunofluorescence (IF) showing the expression of Col2. The results of the quantitative analysis are presented in (**i**). Scale bar, 50 μm (*n* = 6 per group). The data are presented as the mean ± SD, and one-way ANOVA with Tukey’s multiple comparison test was used for statistical analysis (*P < 0.05, **P < 0.01, ***P < 0.005, ****P < 0.001).

KIT^W-SH/W-SH^ mice reportedly exhibit multiple physical defects, such as anemia and iron deficiency, in addition to mast cell defects; therefore, we next investigated the contribution of mast cells to Modic changes through the use of specific micromolecules. Compound 48/80 is a widely utilized mast cell activator^39^, while cromolyn acts as a mast cell stabilizer^40^. Using micro-CT and MRI, as well as Safranin O/Fast Green staining, we did not observe any significant differences among the four groups after 2 weeks, suggesting that Modic changes require a certain amount of time to develop (Supplementary Fig. 2a–g). However, after 4 weeks, the modeling group exhibited more pronounced Modic changes than did the control group, showing decreased T2 signal intensity, vertebral endplate defects, and bone destruction. Administration of cromolyn alleviated Modic changes, while compound 48/80 significantly exacerbated Modic changes (Fig. 3a, b). Further morphological analysis revealed that cromolyn reduced Modic changes, with less nucleus pulposus loss, fewer cartilage defects and less bone destruction, whereas compound 48/80 promoted the progression of Modic changes, especially increased bone destruction and increased histological scores (Fig. 3c–e and Supplementary Fig. 2h).

Then, we assessed the expression of Col2, Sox9, and MMP13. Cromolyn resulted in increased expression of Sox9 and Col2 compared to that in the model group, while it inhibited MMP13 expression. Treatment with compound 48/80 promoted MMP13 expression and decreased Col2 and Sox9 expression compared to those in the model group (Fig. 3f–i, Supplementary Fig. 2i, j). Thus, the pivotal role of mast cell activation in the development of Modic changes is evident.

### Mrgprb2 contributed to mast cell activation in Modic changes

**Fig. 4:**
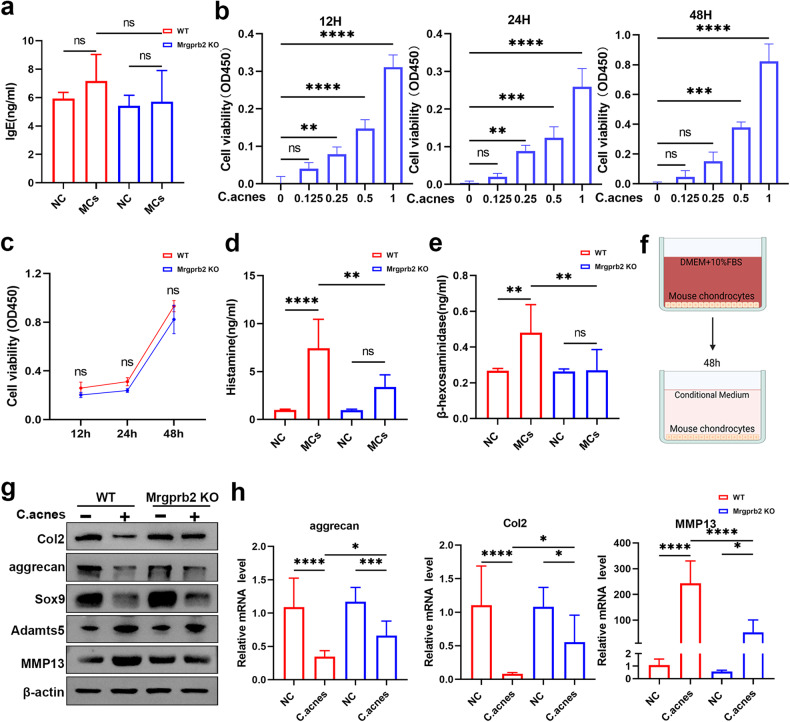
Mrgprb2 contributed to mast cell activation in Modic changes. **a** Serum IgE levels were assessed by ELISA in WT and Mrgprb2 KO mice with or without Modic changes for 4 weeks (*n* = 6 per group). **b** Relative cell viability assays (CCK-8) of primary Mrgprb2-deficient mouse mast cells stimulated with *C. acnes* (1 × 10^7^ CFU/ml) (*n* = 4 per group). **c** Relative cell viability assays (CCK-8) of WT mast cells and Mrgprb2-deficient mast cells treated with *C. acnes* (1 × 10^7^ CFU/ml) (*n* = 4 per group). **d**, **e** ELISA analysis of histamine and β-hexosaminidase in the sera of WT and Mrgprb2-KO mice with or without Modic changes for 4 weeks (*n* = 6 per group). **f** Primary chondrocytes obtained from WT mice and Mrgprb2 KO mice were exposed to conditioned medium obtained from the supernatant of the aforementioned experiments for 48 h. **g** Western blot analysis of the expression of Col2, aggrecan, Sox9, Adamts5, MMP13, and β-actin. **h** The relative mRNA levels of marker genes were determined by reverse transcription‒polymerase chain reaction (RT‒PCR). The results presented in this study are based on data obtained from a minimum of three independent experiments. The data are presented as the mean ± SD, and one-way ANOVA with Tukey’s multiple comparison test was used for statistical analysis (*P < 0.05, **P < 0.01, ***P < 0.005, ****P < 0.001).

To gain a deeper understanding of the underlying mechanism of mast cell activation in Modic changes, we first examined the level of IgE, the classic molecule that activates mast cells, but no significant difference was found between the NC and Modic change groups (Fig. 4a). Recent studies have demonstrated that a new receptor, Mrgprb2, is involved in activating mast cells^41^. We suspected that this receptor was responsible for mast cell activation in Modic changes. To test our hypothesis, we isolated mast cells from Mrgprb2 KO mice and stimulated them with *C. acnes*. Intriguingly, Mrgprb2 knockout did not decisively affect the viability of mast cells (Fig. 4b, c). Toluidine blue staining revealed that there were no significant alterations in cell volume or cytoplasmic granules among Mrgprb2-deficient mast cells (Supplementary Fig. 3a). Next, we generated a model of Modic changes in both WT and Mrgprb2 KO mice and analyzed the serum histamine and β-hexosaminidase levels. We found that Modic change modeling significantly upregulated histamine and β-hexosaminidase in WT mice, but this increase was attenuated in Mrgprb2 KO mice (Fig. 4d, e). These data indicated that the specific receptor Mrgprb2 contributed to mast cell activation in Modic changes.

Then, we collected conditioned medium from cocultured mast cells treated with or without *C. acnes* and performed coculture experiments with chondrocytes. Exposure to *C. acnes*-treated conditioned medium decreased the expression of Col2, aggrecan, and Sox9 and increased the expression of Adamts5 and MMP13 in chondrocytes (Fig. 4f–h). However, conditioned medium from the Mrgprb2 KO group had a similar effect on chondrocytes, but the decreases in Col2, aggrecan, and Sox9 and the increases in Adamts5 and MMP13 were substantially reduced. These findings suggested that mast cells activated by Mrgprb2 affect chondrocyte matrix metabolism and subsequently contribute to Modic changes.

### Mast cell activation via Mrgprb2 had an impact on chondrocyte matrix metabolism

**Fig. 5:**
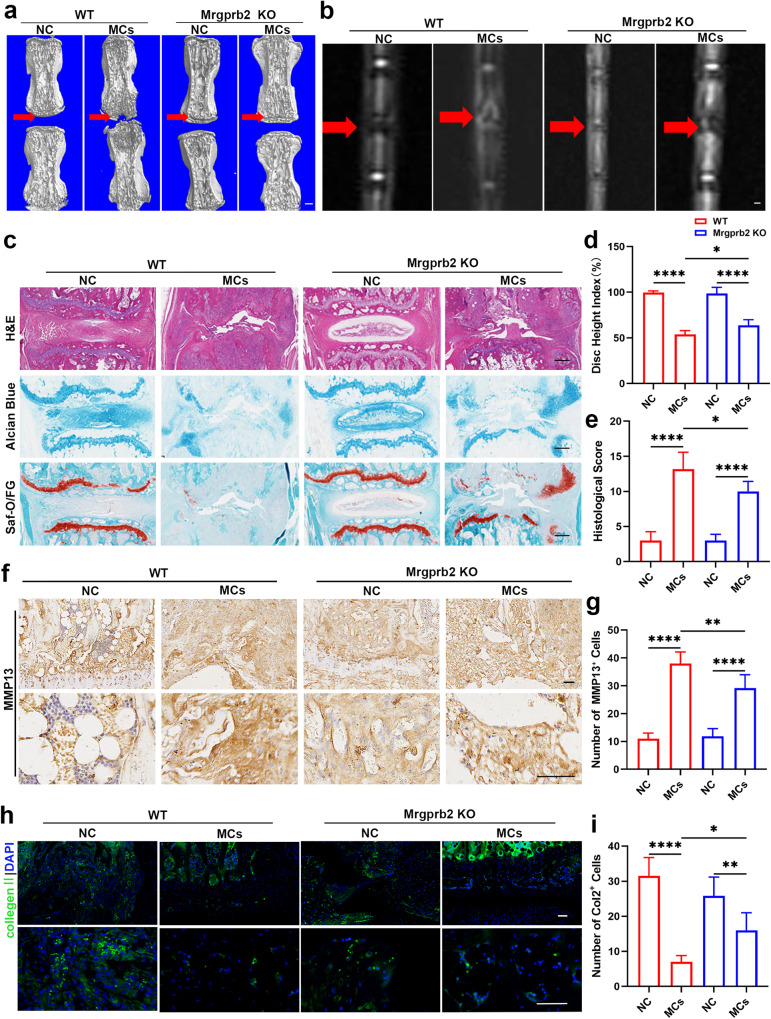
Activation of mast cells via Mrgprb2 affected the metabolism of chondrocytes. **a** Three-dimensional reconstruction of the micro-CT image (*n* = 6 per group). **b** Representative T2-weighted images of caudal discs were obtained from WT mice and Mrgprb2-KO mice with or without Modic changes for 4 weeks (*n* = 6 per group). Scale bar, 200 μm. **c** Representative images of H&E, Safranin O/Fast Green, and Alcian blue staining from WT mice and Mrgprb2-KO mice with or without Modic change modeling for 4 weeks. Scale bar, 50 μm. **d** The disc height indices of the different groups (*n* = 6 per group). **e** Histological scores of the different groups (*n* = 6 per group). **f** Immunohistochemistry (IHC) was used to determine the expression of MMP13. Scale bar, 50 μm. The results of the quantitative analysis are presented in (**g**) (*n* = 6 per group). **h** Immunofluorescence (IF) showing the expression of Col2. Scale bar, 50 μm. The results of the quantitative analysis are presented in (**i**) (*n* = 6 per group). The data are presented as the mean ± SD, and one-way ANOVA with Tukey’s multiple comparison test was used for statistical analysis. (*P < 0.05, **P < 0.01, ***P < 0.005, ****P < 0.001).

To further elucidate the role of Mrgprb2 in mast cells during Modic changes, we generated a model of Modic changes and performed micro-CT and MRI scans on both Mrgprb2 KO and WT mice after 4 weeks. After modeling, Mrgprb2 KO mice displayed greater T2 signals, less endplate destruction and less bone loss, while WT mice exhibited more severe T2 signal reduction, cartilage damage and bone destruction (Fig. 5a, b). H&E, Safranin O/Fast Green, and Alcian blue staining confirmed that Mrgprb2 KO mice had less severe cartilage defects in specific regions than did WT mice after Modic changes were induced (Fig. 5c–e). Immunohistochemistry and immunofluorescence showed that the expression of Col2 and Sox9 decreased obviously and the expression of MMP13 increased significantly in the WT mice, while in the Mrgprb2-KO mice, the decreases in the expression of Col2 and Sox9 and the increase in the expression of MMP13 were less obvious (Fig. 5f–i, Supplementary Fig. 3b, c).

### The activation of mast cells by Mrgprb2 resulted in the aberrant activation of signaling pathways

To elucidate the underlying mechanism by which Mrgprb2 regulates the immune response to *C. acnes* in mast cells, we first analyzed the transcriptome of WT mast cells stimulated with *C. acnes*. Overall Gene Ontology (GO) analysis of the differentially expressed genes (DEGs) revealed that the stimulated WT mast cells were enriched mainly in immune response-related pathways such as the innate immune response, positive regulation of response to external stimulus, and leukocyte migration (Supplementary Fig. 4a, c). Protein‒protein interaction enrichment analysis was performed, and the resulting MCODE networks for each gene list were collected and are presented in Supplementary Fig. 5. The MCODE network in the head was mainly enriched in cell‒cell adhesion, indicating that mast cells may mainly migrate and recruit macrophages to mediate the immune response to *C. acnes* stimulation (Supplementary Fig. 5). Interestingly, the differentially expressed genes (DEGs) of Mrgprb2-deficient mast cells stimulated with or without *C. acnes* were involved in other protective regulatory pathways, such as negative regulation of peptidase activity, regulation of phagocytosis and extracellular matrix organization (Supplementary Fig. 4b, d). The MCODE network in the head was mainly enriched for degradation of the extracellular matrix, suggesting that the migration of Mrgprb2-deficient mast cells stimulated by *C. acnes* was affected (Supplementary Fig. 6).

**Fig. 6:**
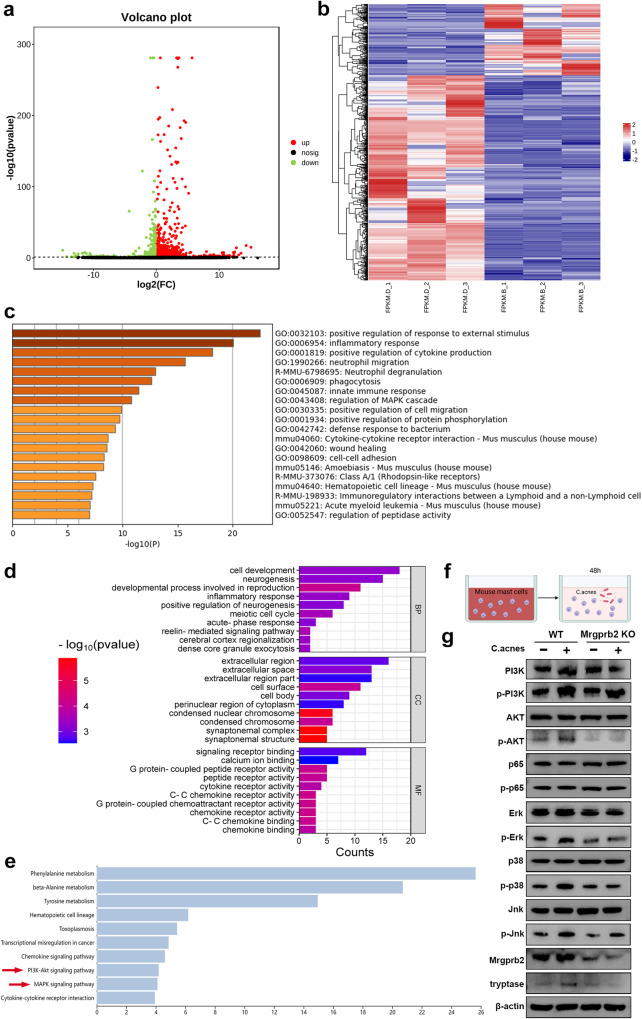
Transcriptome RNA-seq of mast cells from WT and Mrgprb2 KO mice. **a** The volcano plot illustrates the distribution of DEGs (*n* = 3 per group). **b** Heatmap of DEGs in activated mast cells from WT and Mrgprb2 KO mice stimulated with *C. acnes* (*n* = 3 per group). **c** GO enrichment heatmap of overall DEGs (*n* = 3 per group). **d** GO enrichment analysis was performed to evaluate the functional enrichment of downregulated DEGs (*n* = 3 per group). **e** KEGG enrichment analysis was conducted to examine the enriched pathways among the downregulated DEGs (*n* = 3 per group). **f**, **g** Western blot analysis of the expression of PI3K, p-PI3K, AKT, p-AKT, p65, p-p65, Erk, p-Erk, p38, p-p38, JNK, p-JNK, Mrgprb2, tryptase and β-actin during a 48-h coculture of mast cells obtained from WT mice and Mrgprb2 KO mice with *C. acnes*. The results presented in this study are based on data obtained from a minimum of three independent experiments.

Then, we analyzed the DEGs between WT and Mrgprb2-deficient mast cells after *C. acnes* stimulation. A total of 706 DEGs were identified, consisting of 523 upregulated genes and 183 downregulated genes (Fig. 6a, b). GO analysis revealed that the DEGs were enriched in biological processes related to positive regulation of response to external stimulus, inflammatory response, and positive regulation of cytokine production, suggesting that the immune-related function of mast cells in response to *C. acnes* was significantly altered after Mrgprb2 knockout (Fig. 6c). Further analysis suggested that the downregulated genes were enriched in cell development, the extracellular region and signaling receptor binding (Fig. 6d). Notably, some signaling pathways, such as the PI3K-AKT and MAPK pathways, were inhibited (Fig. 6e). To further verify the activation of signaling pathways, we cultured mast cells isolated from both Mrgprb2 KO mice and WT mice, followed by *C. acnes* stimulation. We observed notable activation of the PI3K-AKT, Erk, p38, and JNK pathways in mast cells from WT mice, while p65 exhibited no considerable changes. Additionally, the level of tryptase in WT mast cells notably increased, but there was no significant difference in the level of tryptase in Mrgprb2 KO mast cells, indicating a lack of activation (Fig. 6f, g).

There were also some upregulated genes related to the immune response, cell surface receptor activity, and cytokine activities (Supplementary Fig. 7a, b). Moreover, the transcription factor target prediction of DEGs in the PaGenBase database showed that mast cells stimulated by *C. acnes* might have potential regulatory effects on bone and osteoclasts (Supplementary Fig. 7c). The core MCODE in the PPI network was mainly enriched in the RAP1 signaling pathway and MAPK cascade, indicating that after Mrgprb2 knockout, the loss of Mrgprb2 likely altered the activation of these pathways in mast cells upon *C. acnes* stimulation (Supplementary Fig. 8).

### Activation of mast cells via Mrgprb2 regulated the immune niches involved in Modic changes by modulating macrophage polarization

**Fig. 7:**
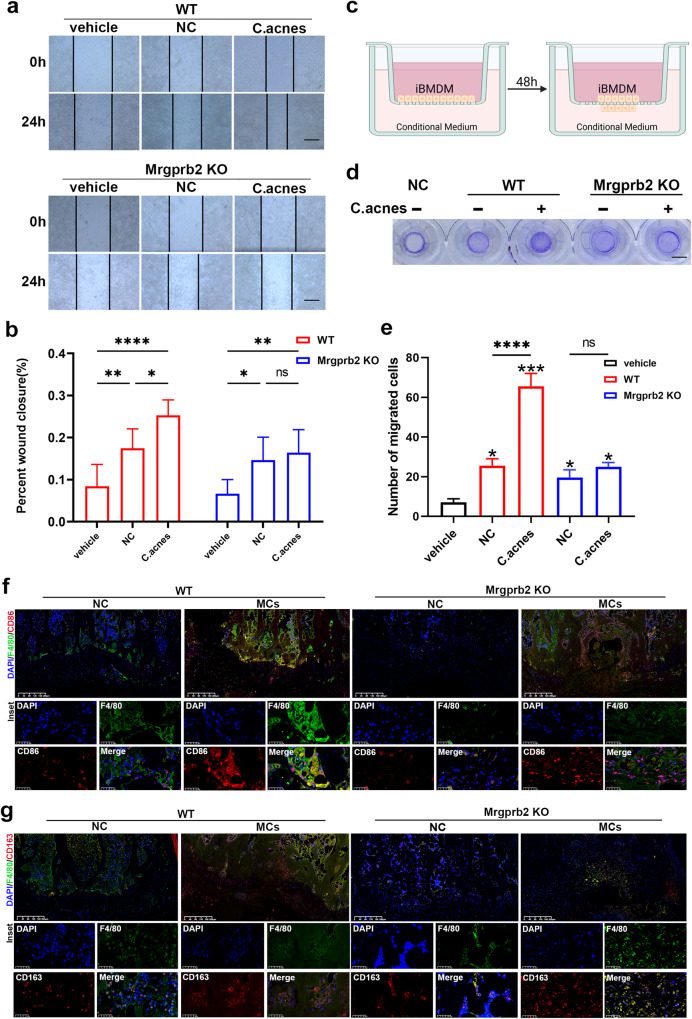
Activation of mast cells via Mrgprb2 regulated the immune niches involved in Modic changes by modulating macrophage polarization. **a** Light microscopy images of RAW264.7 cells cultured with conditioned medium 0 and 24 h after scratch wounding. Quantification of wound closure is shown in (**b**) (*n* = 6 per group). Scale bar, 200 μm. **c**, **d** iBMDM migration was assessed via a Transwell assay, and the quantification of migrated cells is shown in (**e**) (*n* = 6 per group). Scale bar, 500 μm. **f**, **g** Immunofluorescence staining was used to label macrophages (F4/80), M1 macrophages (CD86), M2 macrophages (CD163), and nuclei (DAPI) from WT mice and Mrgprb2 KO mice with or without Modic change modeling for 4 weeks (*n* = 6 per group). Scale bar, 200 μm. The data are presented as the mean ± SD, and one-way ANOVA with Tukey’s multiple comparison test was used for statistical analysis (*P < 0.05, **P < 0.01, ***P < 0.005, ****P < 0.001).

Current research has indicated a strong correlation between mast cells and macrophages, prompting us to investigate the underlying mechanisms in greater detail. We generated conditioned medium by coculturing mast cells with or without *C. acnes* and conducted scratch assays using RAW264.7 cells. Furthermore, we performed Transwell migration assays using iBMDMs. Our results demonstrated that conditioned medium derived from activated mast cells from WT mice significantly enhanced macrophage migration, while conditioned medium derived from activated Mrgprb2 KO mast cells markedly attenuated this recruitment (Fig. 7a–e).

Moreover, we conducted immunofluorescence staining to assess M1 and M2 macrophage polarization in tissue sections obtained from both groups. We observed a significant increase in M1 macrophages within the Modic change areas of WT mice, while no significant changes were found in M2 macrophages. Conversely, we noted a marked increase in M2 macrophages but not M1 macrophages within the Modic changes areas of Mrgprb2 KO mice (Fig. 7f, g). Thus, our findings suggested that the activation of mast cells through Mrgprb2 was a key factor in the pathogenesis of Modic changes, as Mrgprb2 recruited macrophages and modulated their polarization.

### Mast cell reconstitution significantly affected the severity of Modic changes

**Fig. 8:**
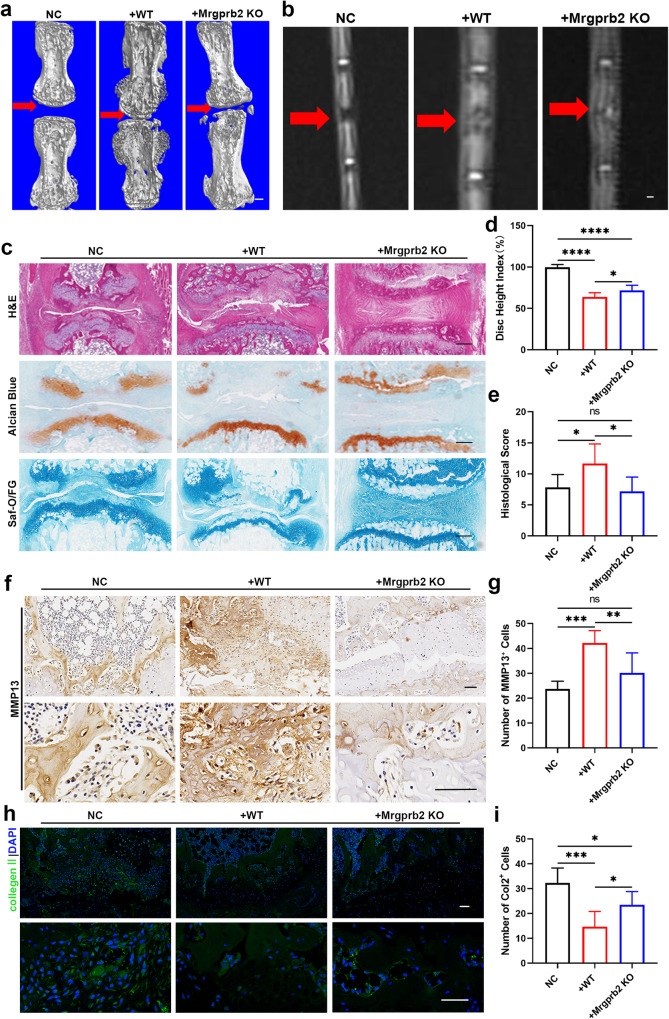
Mast cell reconstitution significantly affects the severity of Modic changes. All KIT^W-SH/W-SH^ mice were subjected to Modic change modeling and randomly divided into three groups: the NC group, the WT mast cell tail vein infusion group and the Mrgprb2-deficient mast cell tail vein infusion group (*n* = 6 per group). **a** Three-dimensional reconstruction of micro-CT analysis from different groups. Scale bar, 200 μm. **b** T2-weighted images of caudal segments from different groups. Scale bar, 200 μm. **c** Representative images of H&E, Safranin O/Fast Green, and Alcian blue staining from different groups. Scale bar, 50 μm. **d** Disc height index from different groups. **e** Histological scores from different groups. **f** Immunohistochemistry (IHC), the expression of MMP13. Quantitative analysis is presented in (**h**). Scale bar, 50 μm. **g** Immunofluorescence (IF), the expression of Col2. Quantitative analysis is presented in (**i**). Scale bar, 50 μm. Data are presented as the mean ± SD, the one-way ANOVA with the Tukey’s multiple comparison test were used for statistical analysis (*P < 0.05, **P < 0.01, ***P < 0.005, ****P < 0.001).

To further verify our conclusions, we subjected KIT^W-SH/W-SH^ mice to mast cell refusion via tail vein injection and generated a model of Modic changes. After 4 weeks, micro-CT and MRI showed that the group with Mrgprb2-deficient mast cell refusion exhibited less T2 signal reduction, less severe endplate defects and less bone destruction, while the group with WT mast cell refusion showed more severe cartilage destruction and fewer endplate defects than did the vehicle group (Fig. 8a, b). H&E, Safranin O/Fast Green, and Alcian blue staining consistently revealed less cartilage damage in the group with reconstituted Mrgprb2-deficient mast cells than in the group with reconstituted WT mast cells (Fig. 8c–e). Immunohistochemistry and immunofluorescence staining revealed a slight decrease in Col2 and Sox9 expression and a slight increase in MMP13 levels in the group with reconstituted Mrgprb2-deficient mast cells compared to those in the NC group. However, in the group with reconstituted WT mast cells, the expression of Col2 and Sox9 dramatically decreased, and the expression of MMP13 markedly increased (Fig. 8f–i, Supplementary Fig. 9a, b). These findings provide additional evidence supporting the pivotal role of Mrgprb2-activated mast cells in the progression of Modic changes.

## Discussion

Chronic inflammatory conditions, such as inflammatory bowel disease, chronic headaches, skeletal muscle degeneration, and osteoarthritis, involve immune cells^42,43^. Mast cells have attracted attention for their role in innate immunity, but their precise involvement in intervertebral disc degeneration is still unclear^44,45^. Through a comprehensive approach involving cellular experiments and animal models, we identified a previously unrecognized pivotal role of mast cells in both the initiation and progression of Modic changes. Notably, mast cells exert regulatory functions by modulating the aggregation and polarization of macrophages within immune niches, thereby initiating downstream inflammatory cascades^42,46,47^. Moreover, mast cell activation and degranulation significantly affect chondrocyte matrix metabolism, contributing to disc degeneration and facilitating the development of Modic changes.

To confirm the observed phenotype, we utilized Mast cell-deficient KIT^W-SH/W-SH^ mice^48,49^. Our findings demonstrated that KIT^W-SH/W-SH^ mice exhibited milder intervertebral disc degeneration and Modic changes than did WT mice. These results provide further support for the substantial impact of mast cells on Modic changes and emphasize their potential as therapeutic targets for clinical interventions. Compound 48/80 is a strong stimulator of mast cell activation and degranulation, whereas cromolyn functions as an inhibitor^50–52^, suppressing mast cell release of mediators. The intraperitoneal administration of these two compounds to WT mice yielded noteworthy findings. Compared with those in the control group, the mice in the group injected with compound 48/80 showed a significant exacerbation of Modic changes, while those in the group injected with cromolyn exhibited reduced Modic changes. These findings further reinforce the critical role of mast cells in the pathogenesis of Modic changes, highlighting their importance in this pathological process.

Mast cells have many receptors, and MRGPRX is a member of the primate-specific subfamily X of Mas-related G protein-coupled receptors (GPCRs), which are orphan receptors classified within the rhodopsin-like class A GPCR family^53,54^. These receptors are of interest due to their unique expression on sensory neurons and immune cells, as well as their involvement in various physiological processes, such as pain perception, inflammation, immune responses, wound healing, pseudoallergic reactions, and potentially cancer development. Among the MRGPRX receptors, MRGPRX2 is a crucial mast cell receptor involved in anaphylactoid drug reactions and various skin and mucosal disorders, such as urticaria, atopic dermatitis, rosacea, and allergic rhinitis^55–57^. Due to their functional importance, targeting MRGPRX receptors shows promise for the development of novel therapeutic interventions.

The lack of suitable animal models for studying primate-specific receptors has been a major obstacle in this field. However, recent progress in humanized mouse development and the discovery of a mouse ortholog for MRGPRX2, called Mrgprb2, have opened up new avenues for research^52^. Although Mrgprb2 may not fully replicate the functions of its primate counterpart, this molecule still has some similarities that make it easier to study the roles of these receptors under both normal and disease conditions. These advancements provide good opportunities for exploring the functions and potential treatments related to primate-specific receptors in more manageable experimental systems.

Despite extensive research on Mrgprb2 as a mast cell receptor, its involvement in disc degeneration has received limited attention. This study revealed an unrecognized role of mast cells in Modic changes associated with disc degenerative disease, primarily through the activation of Mrgprb2. Compared with their wild-type counterparts, Mrgprb2 knockout mice exhibited a notable decrease in disc degeneration and Modic changes under identical modeling conditions as those of the control group. However, the molecular mechanism associated with Mrgprb2 remains incompletely understood. Therefore, further investigations are warranted to elucidate the intricate molecular pathways underlying Mrgprb2 and its role in disc degeneration. These studies will enhance our understanding of Modic changes and identify potential therapeutic targets.

Macrophages, as well as mast cells, are crucial to innate immunity. These cells have a profound impact on various host defense responses and other biological processes, such as regulating ROS levels, maintaining iron homeostasis, repairing tissues, and maintaining metabolic functions^58,59^. The functional polarization of these cells, either the proinflammatory M1 or immunoregulatory M2 phenotype, is determined by environmental cues^59–61^. Mast cells activated by Mrgprb2 in Modic changes lead to dysregulated macrophage polarization, with an increased predominance of the M1 subtype and a simultaneous decrease in the prevalence of the M2 subtype. This change exacerbates the pathological progression of Modic changes.

Nonetheless, it is crucial to recognize the limitations inherent in our study. We did not explore the downstream signaling pathways activated by Mrgprb2 receptor activation, and the complex mechanisms underlying mast cell-mediated macrophage polarization remain unclear. Further research is warranted to elucidate these molecular mechanisms in greater detail.

### Supplementary information

Supplementary Information
