# Supplementary material for: Mrgprb2-mediated mast cell activation exacerbates Modic changes by regulating immune niches
Source: Exp Mol Med. 2024 May 1;56(5):1178–92. doi: 10.1038/s12276-024-01230-1 (PMC11148035; doi:10.1038/s12276-024-01230-1)
Supplement: Supplementary file 1 — Supplementary Information [file 12276_2024_1230_MOESM1_ESM.pdf]

Supplementary information

Supplementary Table 1

Gene	F (5'-3')	R (5'-3')
β-actin	AGCCATGTACGTAGCCATCC	CTCTCAGCTGTGGTGGTGAA
Aggrecan	TTGCAGACATTGACGAGTGC	TTAGTCCACCCCTCCTCACA
Col2	TGGCTTAGGGCAGAGAGAGA	CGTCGTGCTGTCTCAAGGT
Mmp13	CTTCTGGCACACGCTTTTCC	ATGGGAAACATCAGGGCTCC

Supplementary Table 1. The primer sequences used in the qPCR experiments are provided.

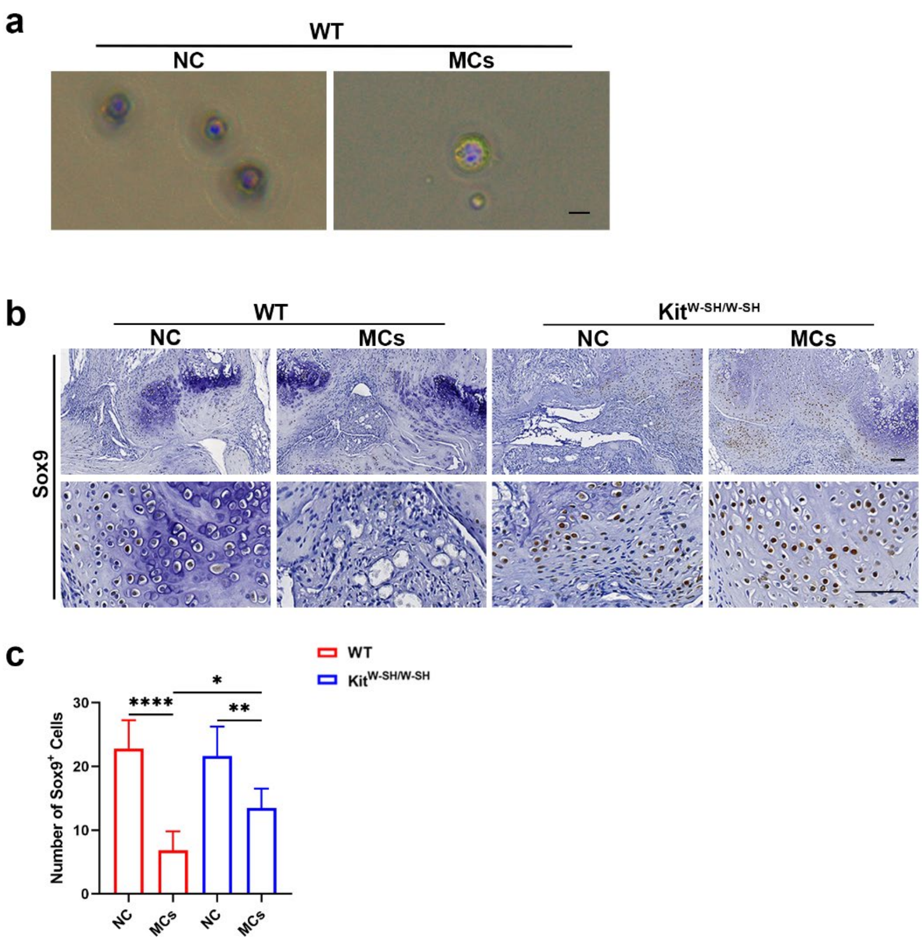

Supplementary Fig. 1. High-microscopy images of mast cells and immunohistochemistry of Sox9.

(a). High-microscopy images of mast cells obtained from WT mice stimulated by C.acnes in vitro. Scale bar, 10 μm.

(b). Immunohistochemistry analysis of Sox9 expression in WT and Kit mice discs with or without Modic changes modeling for 4 weeks. Scale bar, 50 μm. Quantitative analysis presented in (c).

(*P<0.05, ** P<0. 01, ***P<0.005, **** P<0.001).

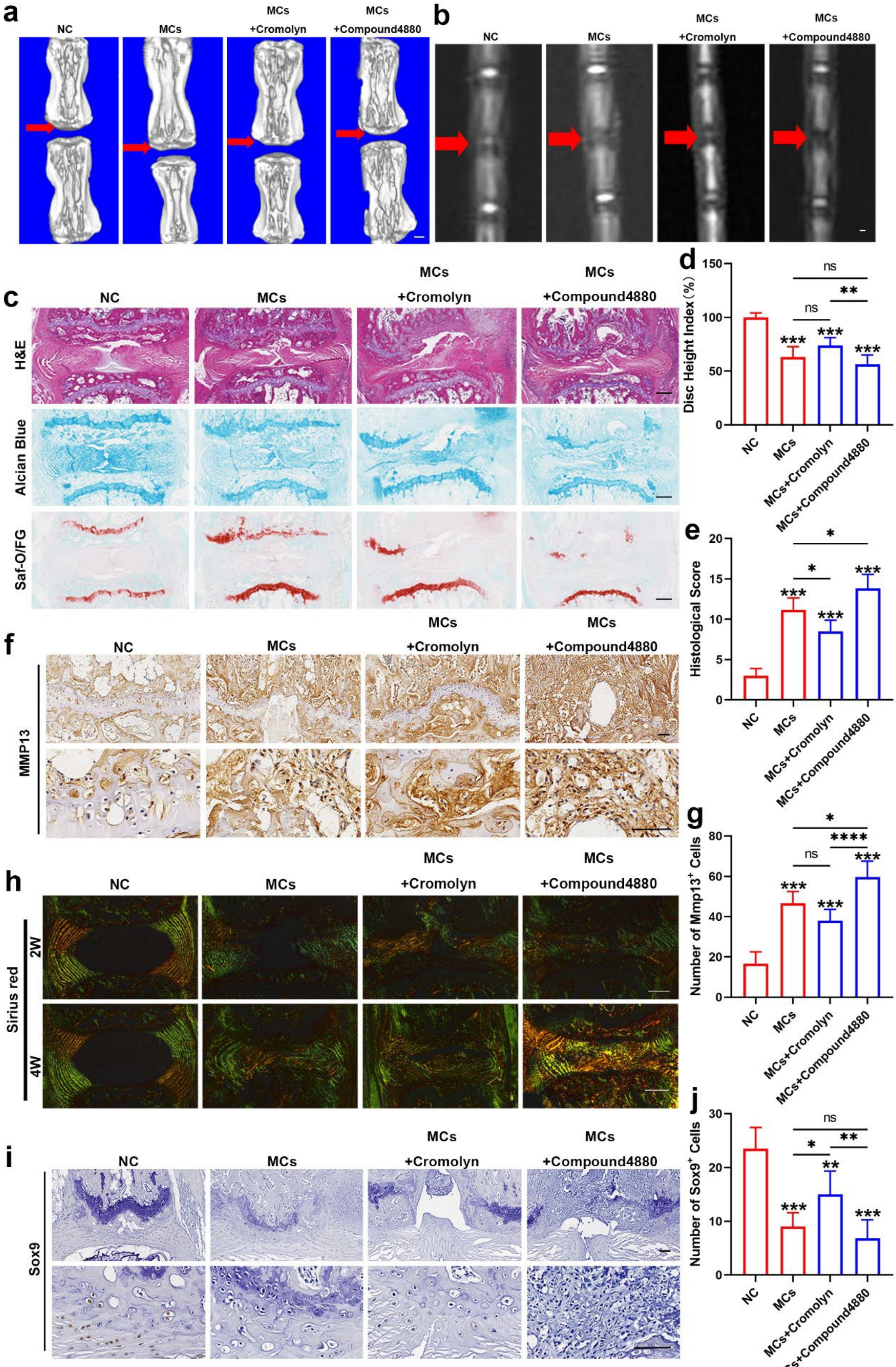

a
NC
MCs
MCs +Cromolyn
MCs +Compound4880
b
NC
MCs
MCs +Cromolyn
MCs +Compound4880
c
NC
MCs
MCs +Cromolyn
MCs +Compound4880
H&E
Alcian Blue
Saf-O/FG
d
Disc Height Index (%)
e
Histological Score
f
NC
MCs
MCs +Cromolyn
MCs +Compound4880
MMP13
g
Number of Mmp13⁺ Cells
h
NC
MCs
MCs +Cromolyn
MCs +Compound4880
Sirius red
2W
4W
i
NC
MCs
MCs +Cromolyn
MCs +Compound4880
Sox9
j
Number of Sox9⁺ Cells
NC
MCs
MCs+Cromolyn
MCs+Compound4880
ns

Supplementary Fig. 2. Data of NC, C.acnes, C.acnes+Cromolyn and C.acnes+Compound4880 groups for 2 weeks.

(a). 3D reconstruction of micro-CT analysis obtained from the NC, C.acnes, C.acnes+Cromolyn and C.acnes+Compound4880 groups for 2 weeks. Scale bar, 200 μm.

(b). Representative T2-weighted images of caudal discs in WT mice were obtained from the NC, C.acnes, C.acnes+Cromolyn and C.acnes+Compound4880 groups for 2 weeks. Scale bar, 200 μm.

(c). Representative images of H&E, Safranin O/Fast green, and Alcian blue staining from the NC, C.acnes, C.acnes+Cromolyn and C.acnes+Compound4880 groups for 2 weeks. Scale bar, 50 μm.

(d). Disc height index from different groups.

(e). Histological scores from different groups.

(f). Immunohistochemistry analysis of Mmp13 expression in WT mice discs obtained from the NC, C.acnes, C.acnes+Cromolyn and C.acnes+Compound4880 groups for 2 weeks. Quantitative analysis is shown in (g). Scale bar, 50 μm.

(h). Sirius red staining of NC, C.acnes, C.acnes+Cromolyn and C.acnes+Compound4880 groups for 2 weeks and 4 weeks. Scale bar, 50 μm.

(i). Immunohistochemistry analysis of Sox9 expression in WT mice discs obtained from the NC, C.acnes, C.acnes+Cromolyn and C.acnes+Compound4880 groups for 4 weeks. Scale bar, 50 μm. Quantitative analysis is presented in (j).

The results presented in this study are based on data obtained from a minimum of three independent experiments Data are presented as mean±SD, the one-way ANOVA with the Tukey's multiple comparison test were used for statistical analysis. (*P<0.05, ** P<0. 01, ***P<0.005, **** P<0.001).

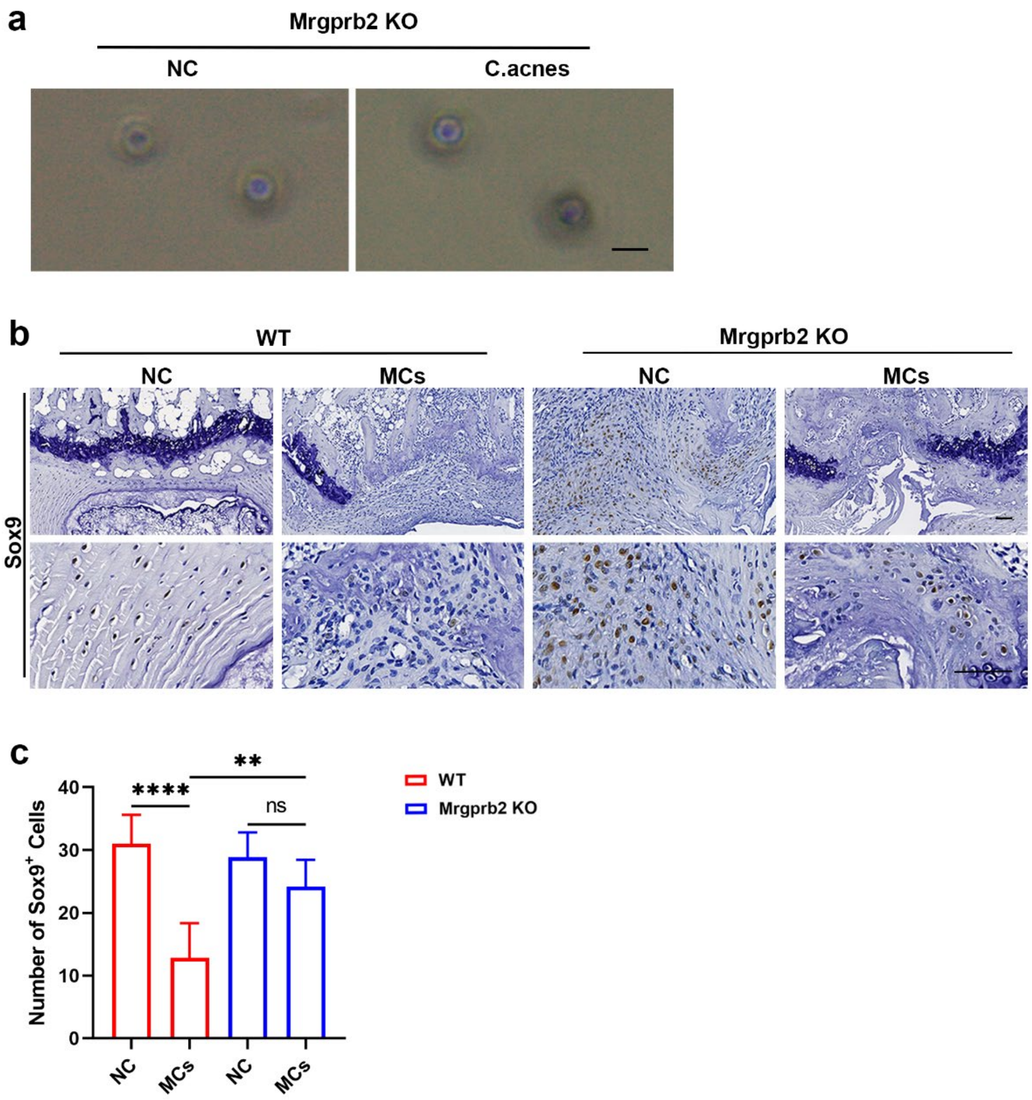

Supplementary Fig. 3. High-microscopy images of mast cells and immunohistochemistry of Sox9.

(a). High-microscopy images of mast cells obtained from WT mice stimulated by C.acnes in vitro. Scale bar, 10 μm. Scale bar, 50 μm.

(b). Immunohistochemistry analysis of Sox9 expression in WT mice discs obtained from WT mice and Mrgprb2 KO mice with or without Modic changes modeling for 4 weeks. Scale bar, 50 μm. Quantitative analysis is shown in (c).

The results presented in this study are based on data obtained from a minimum of three independent experiments Data are presented as mean±SD, the one-way ANOVA with the Tukey's multiple comparison test were used for statistical analysis. (*P<0.05, ** P<0. 01, ***P<0.005, **** P<0.001).

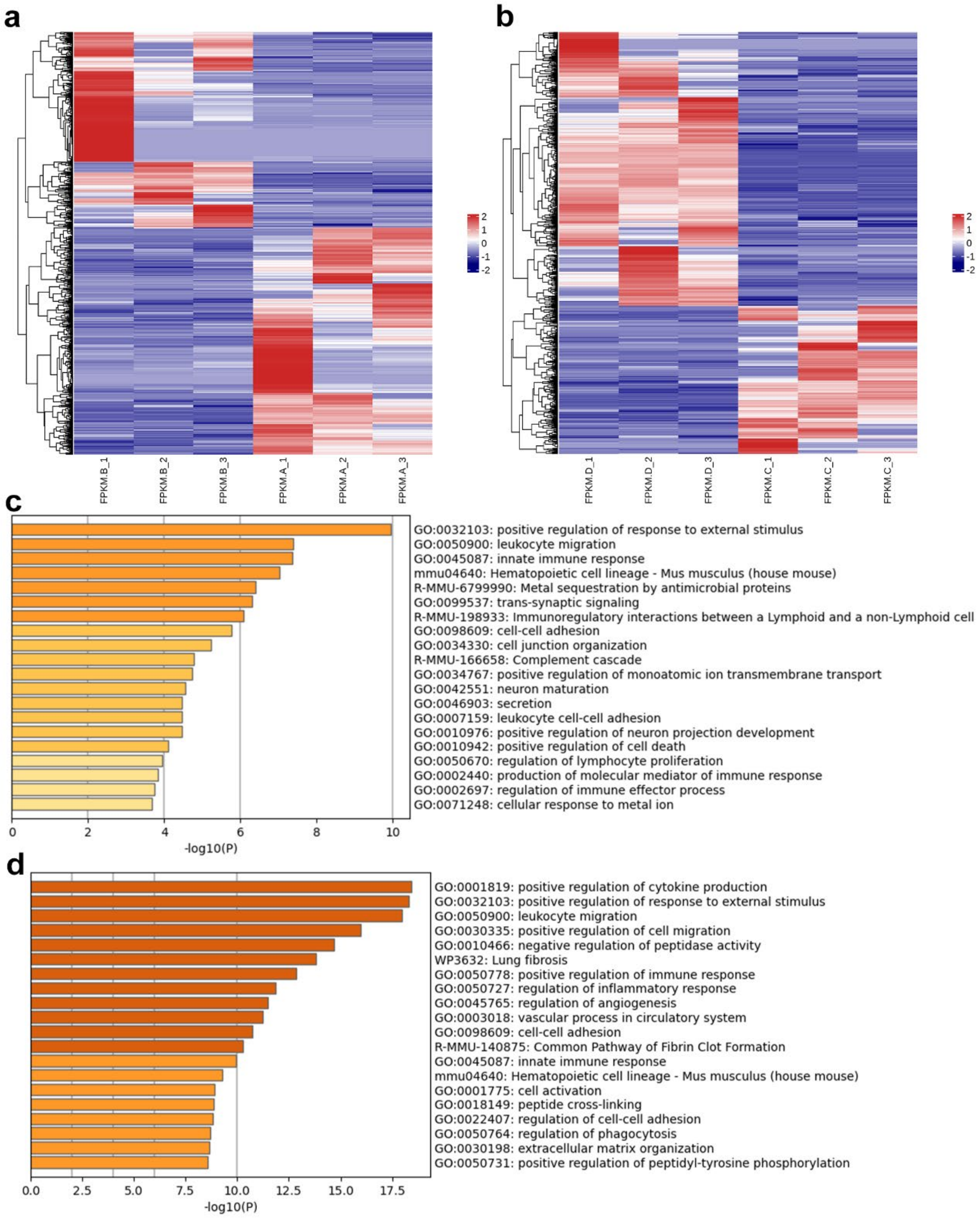

Supplementary Fig. 4. Transcriptome RNAseq of mast cells from WT and Mrgprb2 KO mice.

(a). Heatmap of DEGs of WT mast cells with or without C.acnes stimulation.

(b). Heatmap of DEGs of Mrgprb2-deficient mast cells with or without C.acnes stimulation.

(c). GO enrichment heatmap of overall DEGs of WT mast cells with or without C.acnes stimulation.

(d). GO enrichment heatmap of overall DEGs of Mrgprb2-deficient mast cells with or without C.acnes stimulation.

a

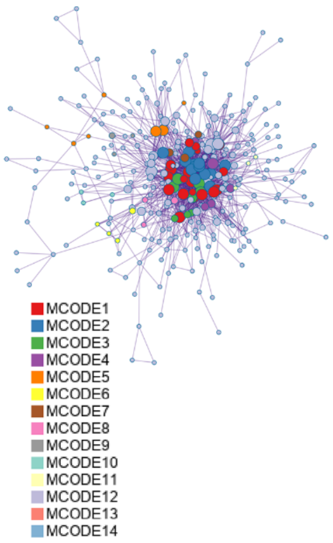

b

Network	Annotation
MCODE_1	GO:0034113|heterotypic cell-cell adhesion|-5.5;GO:0007160|cell-matrix adhesion|-5.3;GO:0033627|cell adhesion mediated by integrin|-5.2
MCODE_2	GO:0007169|transmembrane receptor protein tyrosine kinase signaling pathway|-7.2;GO:0007167|enzyme-linked receptor protein signaling pathway|-5.9;GO:0006468|protein phosphorylation|-5.6
MCODE_3	GO:0006897|endocytosis|-4.7;GO:0006898|receptor-mediated endocytosis|-4.6;mmu04514|Cell adhesion molecules - Mus musculus (house mouse)|-4.5
MCODE_4	GO:0030036|actin cytoskeleton organization|-4.9;GO:0030029|actin filament-based process|-4.7;GO:0007605|sensory perception of sound|-4.1
MCODE_5	GO:0071277|cellular response to calcium ion|-5.6;GO:0051592|response to calcium ion|-5.0;GO:0071248|cellular response to metal ion|-4.7
MCODE_6	R-MMU-173623|Classical antibody-mediated complement activation|-10.0;GO:0098883|synapse pruning|-9.8;GO:0150146|cell junction disassembly|-9.4
MCODE_8	GO:0007411|axon guidance|-5.8;GO:0097485|neuron projection guidance|-5.8;GO:0007409|axonogenesis|-5.2
MCODE_10	R-MMU-380108|Chemokine receptors bind chemokines|-7.8;WP2292|Chemokine signaling pathway|-6.2;R-MMU-375276|Peptide ligand-binding receptors|-6.2
MCODE_11	mmu05033|Nicotine addiction - Mus musculus (house mouse)|-8.2;mmu04727|GABAergic synapse - Mus musculus (house mouse)|-7.1;mmu05032|Morphine addiction - Mus musculus (house mouse)|-7.1
MCODE_13	GO:0043651|linoleic acid metabolic process|-8.5;WP4348|Eicosanoid metabolism via lipoxygenases (LOX)|-8.5;GO:0019369|arachidonic acid metabolic process|-7.3
MCODE_14	GO:0070830|bicellular tight junction assembly|-7.5;GO:0120192|tight junction assembly|-7.4;GO:0120193|tight junction organization|-7.4

c

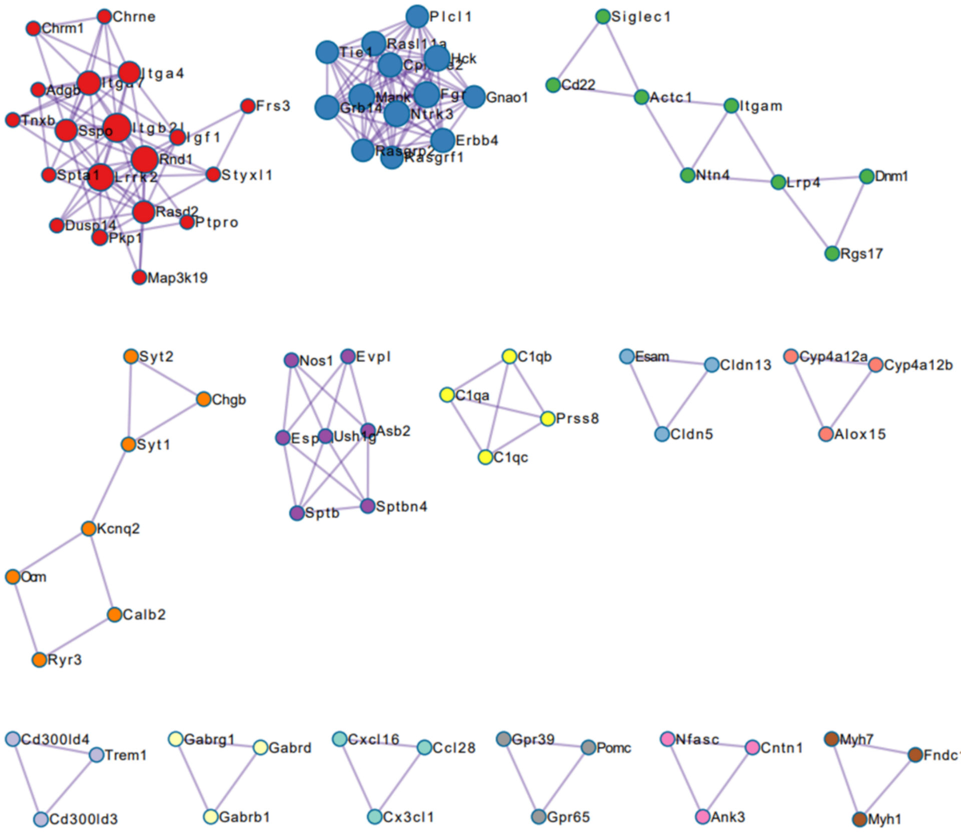

Supplementary Fig. 5. The protein-protein interaction network (PPI) and MCODE components were analyzed based on the differentially expressed gene lists of WT mast cells.

(a). PPI networks of DEGs (WT mast cells stimulated by C.acnes vs control)

(b). The MCODE algorithm was employed to identify highly interconnected network components.

(c). Clusters were assigned different colors based on their ID, indicating that nodes within the same cluster tend to be in close proximity to each other.

a

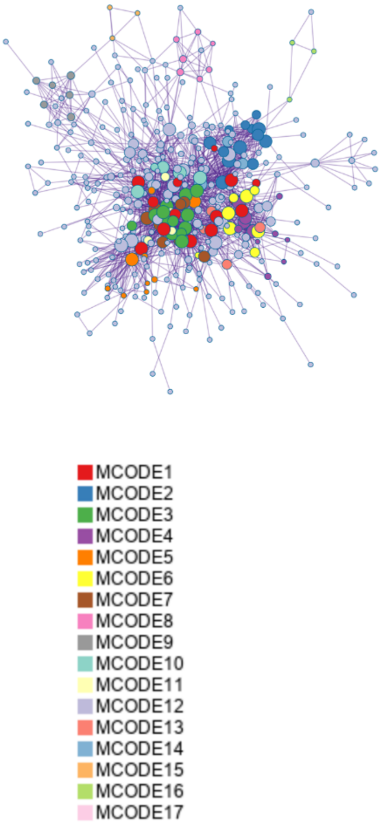

b

Network	Annotation
MCODE_1	R-MMU-1474228|Degradation of the extracellular matrix|-12.6;R-MMU-1474244|Extracellular matrix organization|-12.3;WP441|Matrix metalloproteinases|-11.2
MCODE_2	R-MMU-380108|Chemokine receptors bind chemokines|-34.3;R-MMU-375276|Peptide ligand-binding receptors|-26.9;R-MMU-418594|G alpha (i) signalling events|-24.6
MCODE_3	GO:0043087|regulation of GTPase activity|-5.9;mmu04360|Axon guidance - Mus musculus (house mouse)|-5.4;mmu04015|Rap1 signaling pathway - Mus musculus (house mouse)|-5.2
MCODE_4	R-MMU-2173782|Binding and Uptake of Ligands by Scavenger Receptors|-6.4;R-MMU-5653656|Vesicle-mediated transport|-2.7
MCODE_5	GO:0061760|antifungal innate immune response|-10.3;mmu04625|C-type lectin receptor signaling pathway - Mus musculus (house mouse)|-9.0;R-MMU-5621480|Dectin-2 family|-8.4
MCODE_6	mmu04610|Complement and coagulation cascades - Mus musculus (house mouse)|-12.7;R-MMU-109582|Hemostasis|-10.5;WP460|Blood clotting cascade|-10.4
MCODE_7	WP85|Focal adhesion|-6.4;mmu04510|Focal adhesion - Mus musculus (house mouse)|-6.2;WP2841|Focal adhesion: PI3K-Akt-mTOR signaling pathway|-5.5
MCODE_8	R-MMU-3214858|RMTs methylate histone arginines|-11.1;R-MMU-212300|PRC2 methylates histones and DNA|-10.9;R-MMU-8936459|RUNX1 regulates genes involved in megakaryocyte differentiation and platelet function|-10.8
MCODE_9	mmu00982|Drug metabolism - cytochrome P450 - Mus musculus (house mouse)|-8.7;mmu00980|Metabolism of xenobiotics by cytochrome P450 - Mus musculus (house mouse)|-8.7;mmu00983|Drug metabolism - other enzymes - Mus musculus (house mouse)|-8.3
MCODE_10	mmu05140|Leishmaniasis - Mus musculus (house mouse)|-6.5;GO:0007249|I-kappaB kinase/NF-kappaB signaling|-6.4;mmu05142|Chagas disease - Mus musculus (house mouse)|-5.9
MCODE_11	R-MMU-388841|Costimulation by the CD28 family|-10.3;GO:0045061|thymic T cell selection|-8.2;GO:0045058|T cell selection|-7.2
MCODE_12	R-MMU-190861|Gap junction assembly|-11.4;R-MMU-190828|Gap junction trafficking|-10.9;R-MMU-157858|Gap junction trafficking and regulation|-10.8
MCODE_13	mmu04610|Complement and coagulation cascades - Mus musculus (house mouse)|-9.4;GO:0031639|plasminogen activation|-9.2;GO:0034116|positive regulation of heterotypic cell-cell adhesion|-9.0
MCODE_15	mmu00230|Purine metabolism - Mus musculus (house mouse)|-6.6;WP2185|Purine metabolism|-6.3
MCODE_16	R-MMU-419812|Calcitonin-like ligand receptors|-10.3;R-MMU-373080|Class B/2 (Secretin family receptors)|-7.7;GO:0007189|adenylate cyclase-activating G protein-coupled receptor signaling pathway|-6.6

c

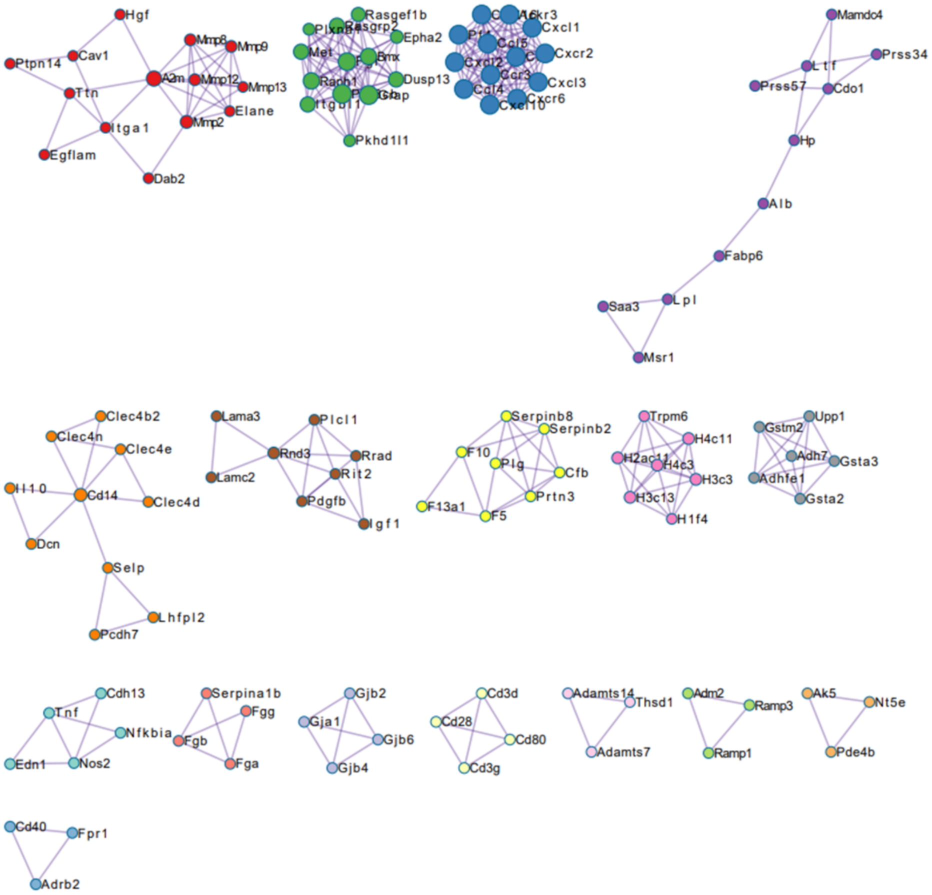

Supplementary Fig. 6. The protein-protein interaction network (PPI) and MCODE components were analyzed based on the differentially expressed gene lists of Mrgprb2 KO mast cells.

(a). PPI networks of DEGs (Mrgprb2 KO mast cells stimulated by C.acnes vs control)

(b). MCODEs has been applied to identify densely connected network components

(c). Clusters were assigned different colors based on their ID, indicating that nodes within the same cluster tend to be in close proximity to each other.

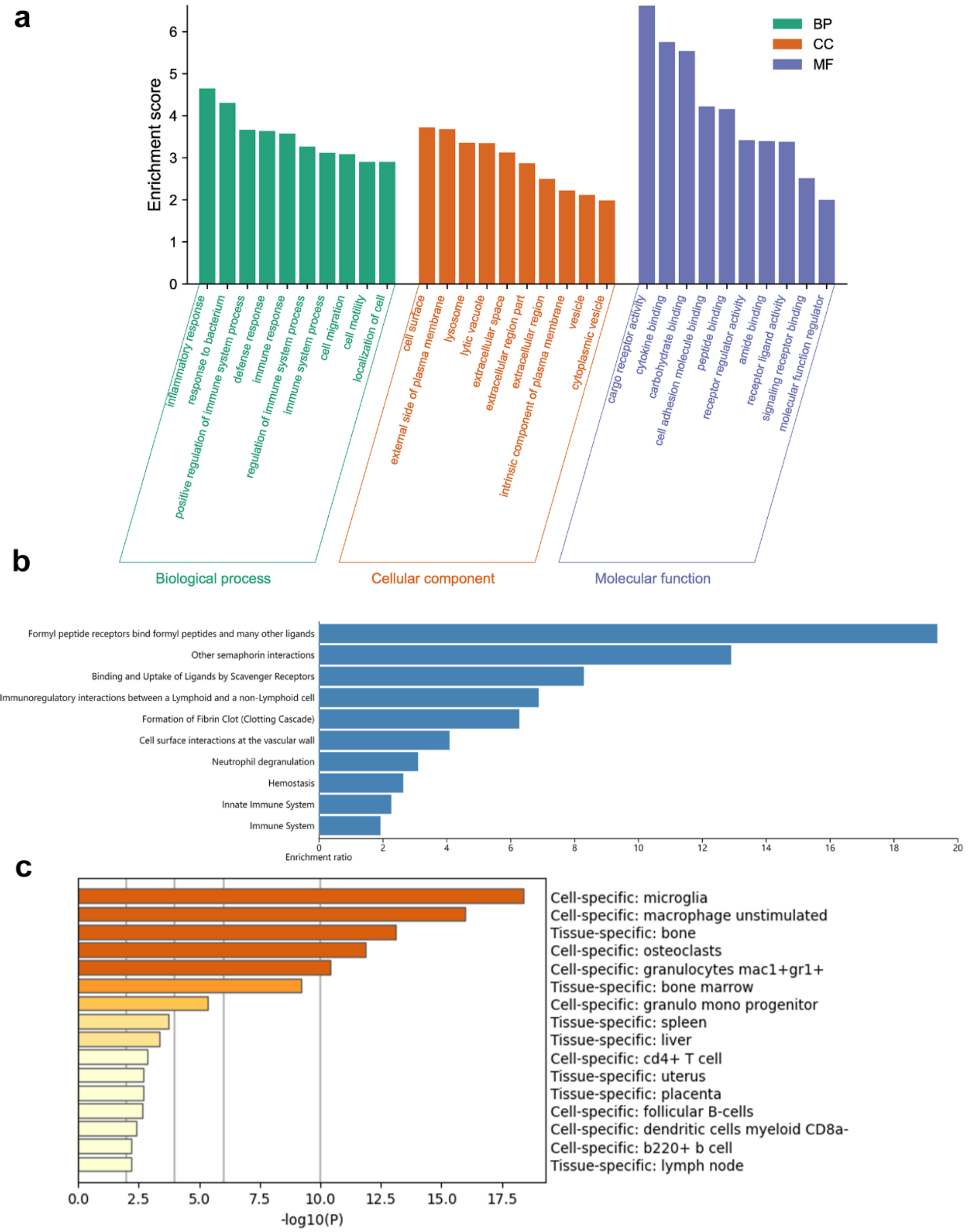

Supplementary Fig. 7. Transcriptome RNAseq of mast cells from WT and Mrgprb2 KO mice.

(a). GO enrichment analysis was performed to assess the functional enrichment of upregulated DEGs.

(b). KEGG enrichment analysis was conducted to examine the enriched pathways among upregulated DEGs.

(c). A summary of enrichment analysis in PaGenBase was generated to investigate the gene regulatory networks related to upregulated DEGs.

a

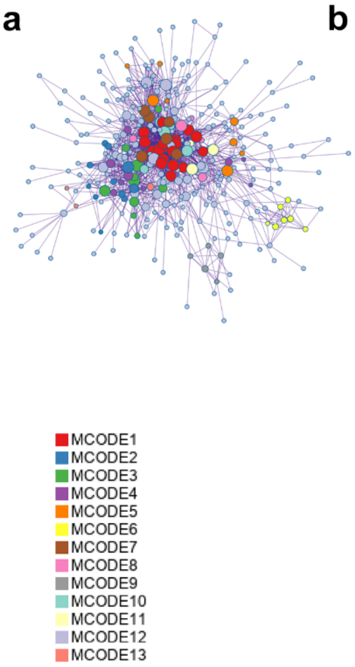

b

Network	Annotation
MCODE_1	mmu04015|Rap1 signaling pathway - Mus musculus (house mouse)|-9.3;GO:0043408|regulation of MAPK cascade|-8.2;GO:0043315|positive regulation of neutrophil degranulation|-7.7
MCODE_2	GO:0010951|negative regulation of endopeptidase activity|-9.6;WP441|Matrix metalloproteinases|-9.0;GO:0010466|negative regulation of peptidase activity|-8.8
MCODE_3	R-MMU-375276|Peptide ligand-binding receptors|-12.3;R-MMU-418594|G alpha (i) signalling events|-11.2;R-MMU-388396|GPCR downstream signalling|-10.9
MCODE_4	GO:0010886|positive regulation of cholesterol storage|-8.7;GO:0010744|positive regulation of macrophage derived foam cell differentiation|-7.6;GO:0010885|regulation of cholesterol storage|-7.1
MCODE_5	R-MMU-5621480|Dectin-2 family|-8.7;mmu05221|Acute myeloid leukemia - Mus musculus (house mouse)|-8.1;GO:0061760|antifungal innate immune response|-7.7
MCODE_6	mmu00982|Drug metabolism - cytochrome P450 - Mus musculus (house mouse)|-11.1;mmu00980|Metabolism of xenobiotics by cytochrome P450 - Mus musculus (house mouse)|-11.0;GO:0006805|xenobiotic metabolic process|-9.8
MCODE_7	GO:0007409|axonogenesis|-3.7;GO:0061564|axon development|-3.5;GO:0031346|positive regulation of cell projection organization|-3.5
MCODE_8	mmu04510|Focal adhesion - Mus musculus (house mouse)|-12.1;mmu04512|ECM-receptor interaction - Mus musculus (house mouse)|-11.2;mmu04151|PI3K-Akt signaling pathway - Mus musculus (house mouse)|-10.6
MCODE_9	R-MMU-3214858|RMTs methylate histone arginines|-9.2;R-MMU-212300|PRC2 methylates histones and DNA|-9.0;R-MMU-8936459|RUNX1 regulates genes involved in megakaryocyte differentiation and platelet function|-9.0
MCODE_10	GO:0048667|cell morphogenesis involved in neuron differentiation|-5.8;GO:0048812|neuron projection morphogenesis|-5.7;GO:0120039|plasma membrane bounded cell projection morphogenesis|-5.7
MCODE_11	GO:0045862|positive regulation of proteolysis|-4.6;GO:0030162|regulation of proteolysis|-3.7
MCODE_12	R-MMU-168898|Toll-like Receptor Cascades|-6.5;GO:0031349|positive regulation of defense response|-5.1;GO:0006954|inflammatory response|-4.7

c

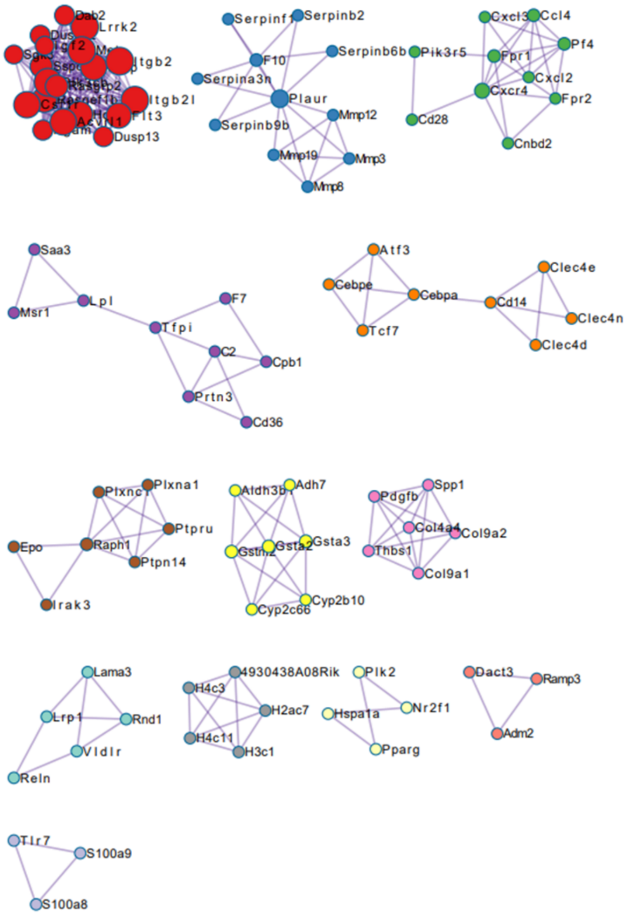

Supplementary Fig. 8. The protein-protein interaction network (PPI) and MCODE components were analyzed based on the differentially expressed gene lists of WT mast cells and Mrgprb2 KO cells stimulated by C.acnes.

(a). PPI networks of DEGs (WT mast cells stimulated by C.acnes vs Mrgprb2 KO mast

cells stimulated by C.acnes)

(b). The MCODE algorithm was employed to identify highly interconnected network components.

(c). Clusters were assigned different colors based on their ID, indicating that nodes within the same cluster tend to be in close proximity to each other.

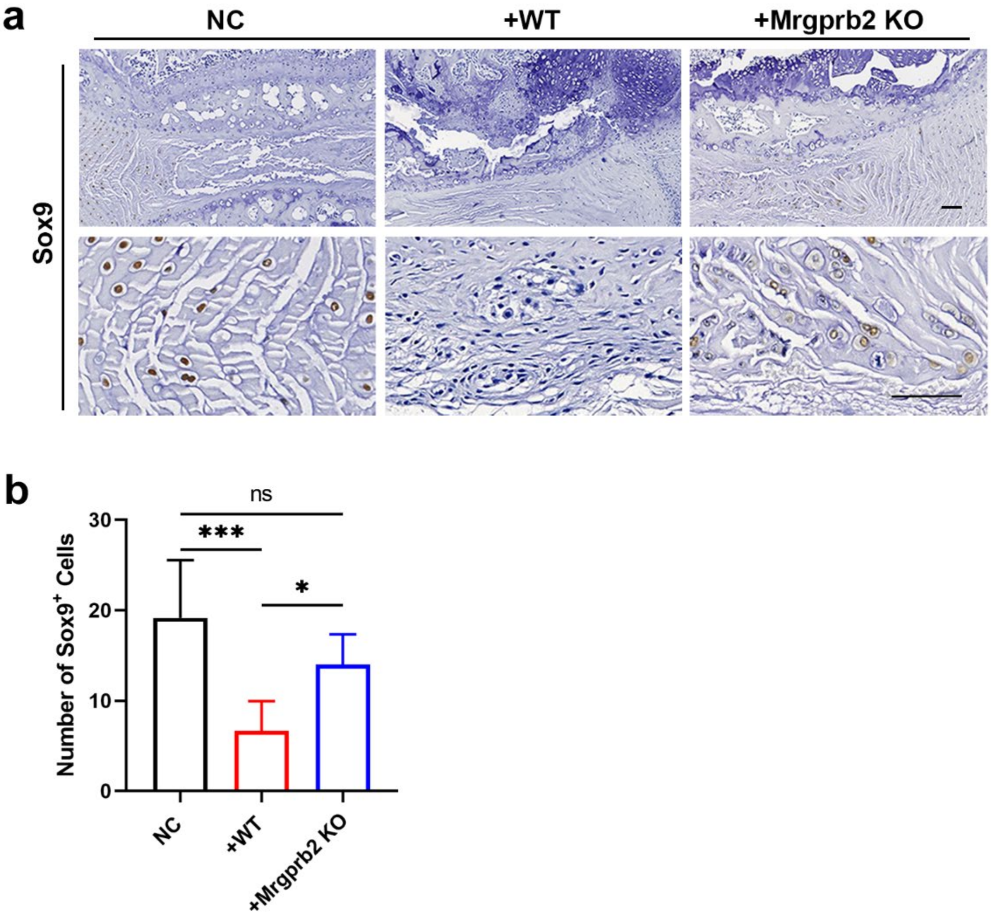

Supplementary Fig. 9. Immunohistochemistry of Sox9.

(a). Immunohistochemistry analysis of Sox9 expression in Kit mice were obtained from the NC, WT mast cells refusion, Mrgpeb2 KO mast cells refusion for 4 weeks. Scale bar, 50 µm. Quantitative analysis is presented in (b).

The presented results were obtained from a minimum of three independent experiments and are presented as the mean ± SD. Statistical significance was indicated as *P<0.05, **P<0.01, ***P<0.005, and ****P<0.001.
